# Forward Osmosis Membrane: Review of Fabrication, Modification, Challenges and Potential

**DOI:** 10.3390/membranes13040379

**Published:** 2023-03-26

**Authors:** Bakr M. Ibraheem, Saif Al Aani, Alanood A. Alsarayreh, Qusay F. Alsalhy, Issam K. Salih

**Affiliations:** 1 Membrane Technology Research Unit, Department of Chemical Engineering, University of Technology-Iraq, Alsinaa Street 52, Baghdad 10066, Iraq; 2 The State Company of Energy Production—Middle Region, Ministry of Electricity, Baghdad 10013, Iraq; 3 Department of Chemical Engineering, Faculty of Engineering, Mutah University, P.O. Box 7, Karak 61710, Jordan; 4 Department of Chemical Engineering and Petroleum Industries, Al-Mustaqbal University College, Hillah 51001, Iraq

**Keywords:** forward osmosis, FO application, thin film composite membrane, thin film nanocomposite membrane, nanoparticles, draw solution, operating conditions, energy consumption

## Abstract

Forward osmosis (FO) is a low-energy treatment process driven by osmosis to induce the separation of water from dissolved solutes/foulants through the membrane in hydraulic pressure absence while retaining all of these materials on the other side. All these advantages make it an alternative process to reduce the disadvantages of traditional desalination processes. However, several critical fundamentals still require more attention for understanding them, most notably the synthesis of novel membranes that offer a support layer with high flux and an active layer with high water permeability and solute rejection from both solutions at the same time, and a novel draw solution which provides low solute flux, high water flux, and easy regeneration. This work reviews the fundamentals controlling the FO process performance such as the role of the active layer and substrate and advances in the modification of FO membranes utilizing nanomaterials. Then, other aspects that affect the performance of FO are further summarized, including types of draw solutions and the role of operating conditions. Finally, challenges associated with the FO process, such as concentration polarization (CP), membrane fouling, and reverse solute diffusion (RSD) were analyzed by defining their causes and how to mitigate them. Moreover, factors affecting the energy consumption of the FO system were discussed and compared with reverse osmosis (RO). This review will provide in-depth details about FO technology, the issues it faces, and potential solutions to those issues to help the scientific researcher facilitate a full understanding of FO technology.

## 1. Introduction

**Figure 1:**
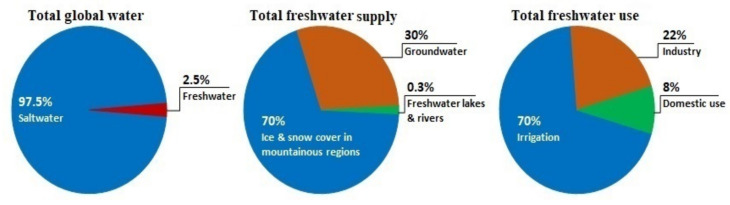
Information about global water and global supply and use of freshwater according to UN-Water “everythingconnects.org/fresh-water (accessed on 25 December 2022)”.

In past decades, freshwater demand has risen substantially owing to population growth, economic development, and different consumption patterns [1]. This ultimately, induced a global clean water scarcity and this scenario will only get worse in the next few decades. According to the United Nations, the lack of safe drinking water will affect nearly 6 billion people by 2050. Figure 1 provides details on global water supply, freshwater supply, and freshwater use according to UN-Water. As a consequence, to address the impending global freshwater shortage problem, sustainable, cost-effective desalination technologies are a necessity to exploit the infinite salty water resources available on the planet [2].

**Figure 2:**
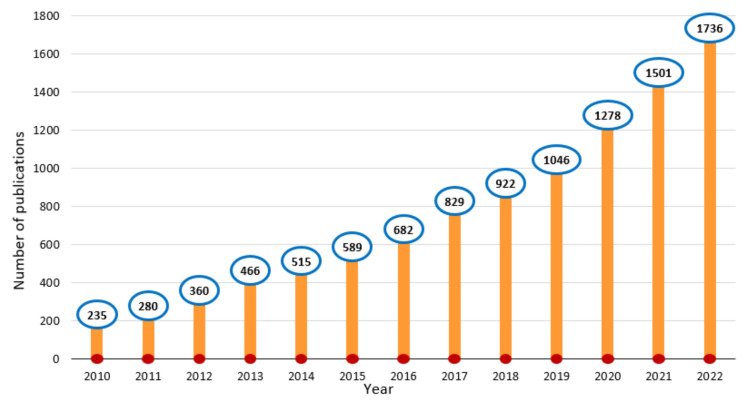
FO publications growth since 2010. The information was obtained from Science Direct using “forward osmosis” as a keyword.

One of these reliable desalination technologies is membrane-based technologies. Membrane technology is gaining increasing popularity in water, wastewater, and many other industrial applications and is divided into (I) pressure-driven membrane processes (PDMPs) such as microfiltration (MF), ultrafiltration (UF), nanofiltration (NF), and reverse osmosis (RO) [3,4,5,6] and (II) osmotically-driven membrane processes (ODMPs) such as forward osmosis (FO) and pressure retarded osmosis (PRO) [6,7]. PDMPs, especially RO, have attracted much attention in water treatment processes due to their high productivity for pure water. Nevertheless, most of these membrane processes are energy-consuming and high-priced [8]. This drawback has led to the investigation of alternate means of desalinating water. ODMPs, especially FO, as an alternative to PDMPs have seen immense attraction within membrane science in recent years [7]. FO as an emerging membrane technology has received tremendous attention recently for its use in water purification [9]. According to Science Direct, academic interest in the FO process has increased in the past twelve years, resulting in enormous document publications on the topic with a total of 10,439 papers published since 2010 (Figure 2) because it has several advantages compared to PDMPs (particularly in RO). Among these advantages, FO can be operated at a low cost because water is driven through the membrane from the feed solution to draw solution by an osmotic pressure gradient rather than hydraulic pressure. This contributes to making the FO process give not only low system energy consumption and strong adaptability but also high water flux and lower membrane fouling by a rejection of a wide range of contaminants [10]. However, the FO process also suffers from various challenges and it is still facing two main challenges related to each other, such as suitable membrane and draw solution [11]. In this regard, researchers have focused on the design/development of FO membranes and draw solutions to reduce FO-related issues (such as concentration polarization, membrane fouling, and reverse solute flux) and make the membrane cleaning procedure easier.

**Figure 3:**
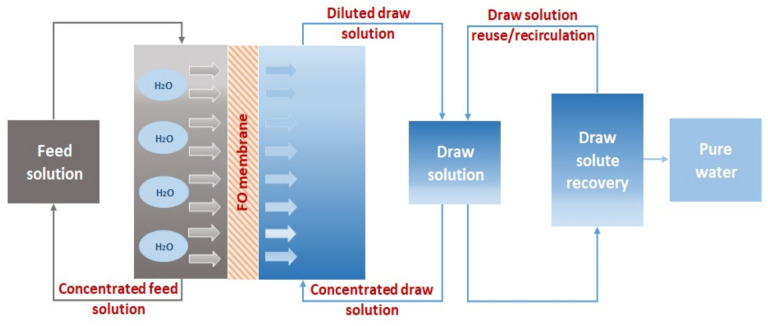
Schematic diagram of a FO process concept.

In practical terms, the FO process operates by concentration (or osmotic pressure) differences between the feed solution (FS), which features a low-salinity solution (i.e., low osmotic pressure), and the draw solution (DS), which feature a high-salinity solution (i.e., high osmotic pressure) [12,13]. As a consequence, FO requires a highly concentrated draw solution to induce the driving force for separation, and hence, the water molecules will start moving from the feed to the draw solution [14,15]. During this process, the majority of the dissolved molecules or multivalent ions already present in the feed water are retained by the membrane. Whereas DS is diluted as water continues to be transported across a membrane, that is, the osmotic pressure of DS decreases and the osmotic pressure of FS increases until it reaches its osmotic equilibrium point. This will determine the final concentration of the dilute DS, which needs an additional process to remove the draw solute and produce pure water, as the product (i.e., dilute DS) cannot be consumed directly as freshwater [15]. A schematic representation of a FO process concept is shown in Figure 3. Thereby, membrane FO and draw solution are certainly the heart of the FO system and play an important role in moving FO forward to commercialization.

According to what was mentioned, FO process efficiency mainly depends on the membrane type and draw solution type. For membrane type, the FO membrane can be divided into two main types: symmetric membranes (i.e., porous and nonporous/dense membranes), and asymmetric membranes (i.e., composite membranes) [16]. Currently, thin-film composite (TFC) membranes are the gold standard for desalinating brackish water (BW) and seawater (SW). Technically, a typical TFC membrane is made from a thin selective layer (10–200 nm thick), precipitated on a macroporous membrane (50–150 μm thick), and backed on non-woven fabric (100–500 μm thick) [17]. Ideally, a membrane that is utilized for a FO process should have a support layer with high flux and an active layer with high water permeability and solute rejection from the FS and the DS at the same time. Moreover, a high reverse solute flux (RSF) (i.e., representing the amount of salt that migrates from the draw solution toward the feed) should be prevented because it would cause serious internal concentration polarization (ICP) and membrane fouling issues throughout an FO process [18]. As to the draw solution type, FO draw solutions can be divided into two basic categories: electrolyte solutions and non-electrolyte solutions. Thus, a key to increasing FO efficiency is choosing DS that features a low RSF, high water flux, and easy regeneration [19]. 

Massive advances in FO applications witnessed recently have attracted the scientific community’s focus on designing high-performance FO membranes along with prominent draw solutes. The prospects for FO technology are booming in many fields that can be summarized in two areas: municipal wastewater, and industry wastewater. First, FO has been applied as a treatment technology for municipal wastewater such as local municipal sewage [20], domestic municipal sewage [21], sludge dewatering [22], secondary and tertiary effluent [23], and landfill leachate [24]. The rejection of nutrients (${\mathrm{NH}}_{4}^{+}$-$N,{\;\mathrm{NO}}_{3}^{-}$-$N,{\;\mathrm{NO}}_{2}^{-}$-N, and${\;\mathrm{PO}}_{4}^{3-}$-P) during the sludge dewatering process was 97%, 90%, 97%, and 99%, respectively [25]. While nutrients rejection from secondary treated effluents was 99.17%, 95.11%, 51.22%, and 97.03%, respectively [26]. Second, FO has been used as a technique for the treatment of industrial wastewater such as the dairy industry [27], leather industry [28], pharmaceutical industry [29], textile industry [30], dye industry [31], pulp and paper industry [32], printed circuit board (PCB) industry [33], and automobile industry [34].

With so many journals, papers, articles, and documents submitted daily on FO technology topics, it is very important for researchers to have a general understanding of the various aspects of the FO process and to keep track of what has been accomplished in many applications. This is where literature reviews come into play a role; these reviews are scientific papers that include the use of findings, ideas, and discussions to analyze general and specific research trends for the FO process. In this regard, this review will shed the light on most important factors that control the performance of the FO process, including the role of both the active layer and support layer and their performance improvement through their modification with nanomaterials, and types of draw solution, as well the role of the operating conditions. Moreover, a detailed discussion regarding the energy consumption (compared to RO) and limitations of the FO process, such as concentration polarization (CP), membrane fouling, and reverse solute diffusion (RSD) is presented by clarifying the factors that affect them and how to mitigate them. In addition, applications are also being identified in the FO process, especially in the field of agriculture such as fertilization or enriched irrigation due to the high water consumption in this field, and also know the potential industrial applications. Finally, case studies and future studies ideas in the FO process were discussed. It is believed that the content of this review provides insight into the full use of the FO process and its associated pros and cons.

## 2. Role of Active Layer and Substrate on FO Performance

The performance of FO membranes is largely determined by the active (selective) layer, which controls water flux, salt rejection, reverse solute flux, and the support (substrate) layer, which determines internal concentration polarization (ICP) grade and effective osmotic gradient of water flux as well as provides mechanical strength and flow channels [35]. For the manufacture of the last layer, the phase inversion method is commonly used to produce films from polymers such as polyvinyl chloride (PVC) [36,37], polysulfone (PSF) [38,39], polyphenylsulfone (PPSU) [39,40], polyether-sulfone (PES) [41,42], and polyacrylonitrile (PAN) [43,44]. The polymer solution is cast on woven and nonwoven backing fabrics at the stage of preparing the membrane substrate. Membranes made from woven fabrics had better mechanical properties, whereas higher pores were observed in membranes manufactured on nonwovens. In addition, nonwoven substrates exhibited better performance in terms of water flux because their structure was thinner and more porous [45]. Moreover, a polyamide (PA) thin film layer is created by the interfacial polymerization (IP) reaction between aromatic amine monomers, such as m-phenylenediamine (MPD), and aromatic acyl chloride monomer, such as trimesoyl chloride (TMC), over the top of the porous substrates [46]. In this regard, the PA layer can also be synthesized using other monomers. The common monomers employed in the manufacture of TFC membranes were reviewed by Farahbakhsh et al. [47]. They include new monomers that can be used in places of MPD, such as p-phenylene diamine (PPD), triethylenetetramine (TETA), piperazine (PIP), and polyethyleneimine (PEI), as well as new monomers that can be used in place of TMC, such as isophthaloyl chloride (IPC), 5-isocyanato-isophthaloyl chloride (ICIC), hyperbranched polyester acyl chloride (HPE-COCl), and tetra-functional biphenyl acyl chloride isomers (mm- BTEC, om-BTEC, and op-BTEC). Likewise, a review of the monomers employed in the fabrication of an active layer of TFC membranes is also included by Li et al. [48]. Indeed, MPD and TMC, which are dissolved separately in aqueous and organic solutions, are the two most popular monomers employed to prepare the separation layer of TFC membranes [46].

Substrates are primarily optimized in terms of hydrophilicity, permeability, and rejection potential by altering fabrication procedures. Factors such as type of solvent, air humidity, processing temperature, polymer concentration, and additives have been demonstrated to affect the properties of supports and, as a result, the performance of TFC membranes [49]. For example, increasing polymer content in solution leads to a higher viscosity, which slows transport rates and delays demixing, as well as produces membranes with thicker top layers, lower porosities, and less macro-void formation [36,38]. Low-concentration PVC (10 wt.%) substrate shows a thinner skin layer, wider channels with thinner walls, a lower amount of finger-like structure, and higher porosity resulting in higher permeability [36]. Whereas the membranes were made using different solvents (dimethylformamide, DMF, and N-methyl-2-pyrrolidinone, NMP) in which a DMF-TFC membrane support surface experienced a greater pore size and porosity, as well as a rougher and more permeable active layer, which increased water and salt permeability. The likelihood of fouling was supposed to be lower, but they found NMP-TFC membrane experienced less flux decrease (7.47 ± 0.15%) than the DMF-TFC membrane (12.7 ± 2.62%) when the feed contained typical organic foulant [50]. Interestingly, adding polyethylene glycol (PEG-400) to the dope solution affects the morphology and properties of the fabricated membranes. At low PEG-400 concentration (6 wt.%), the membrane exhibited better hydrophilicity, higher porosity, and large pores, which feature relatively higher water fluxes and superior salt rejection ability unlike high PEG-400 concentration (9 wt.%) [51]. While the addition of 3 wt.% lithium chloride (LiCl) to the membrane support layer leads to an increase in both the water flux from 3.59 to 6.71, 2.85–6.88, and 3.04–5.72 LMH for PSU, PESU, and PPSU, respectively, and reverse salt flux from 5.33 to 6.84, 4.56–6.86, and 4.95–7.88 gMH for PSU, PESU, and PPSU, respectively, under AL-FS orientation. To increase water flux and minimize reverse salt flux, the membranes must be improved further. Using varied LiCl concentrations in the casting solution, for example [39]. On the other hand, a high air humidity (e.g., 30, 40, and 50%) produced macro-void formation near the surface as well as a structure more prone to defect formation due to increased porosity and decreased mechanical stability of the top layer. Low air humidity (e.g., 20%) caused the formation of a denser and less porous structure with the formation of the largest macro-voids in the upper layer [52]. 

Optimization of substrate layer characteristics is difficult since numerous variables (hydrophilicity, pore size, surface roughness, and even bottom surface structure) are changed, each of which has a different impact on the performance of the final TFC-FO membrane [53]. It has been observed that increasing the content of sulphonated polymer in the membrane substrates stimulated the hydrophilic properties which played an important role in mitigating ICP and improving water flux. It was also found that non-sulfonated polymer supports result in dense bottom surfaces, but membranes from sulfonated materials tend to be porous [54,55]. This approach was confirmed by Han et al. [56] showed that blending a sulfonide polymer (ether ketone) (SPEK) polymer into a PSU substrate for TFC-FO membranes not only plays a major role in forming the whole sponge-like structure but also enhances membrane hydrophilicity and reduces the structure parameter. When 2 M NaCl was used as a draw solution in PRO mode, the TFC-FO membrane containing 50% SPEK in the substrate had the largest water flux of 50 LMH vs. DI water and 22 LMH vs. 3.5 wt% NaCl sample solution. In a similar vein, Corvilain et al. [53] found that addition of a more hydrophilic sulfonated polyetheretherketone polymer (sPEEK) to the polymer solution caused pores in the bottom layer of the support with average pore sizes ranging from 0.07 to 0.30 μm, which enhanced water flux. Using 0.5 M NaCl and deionized (DI) water as the feed pair, TFC membranes containing 5% sPEEK can achieve a water flux of 14.3 LMH under the PRO mode and 6.2 LMH under the FO mode, respectively. 

**Figure 4:**
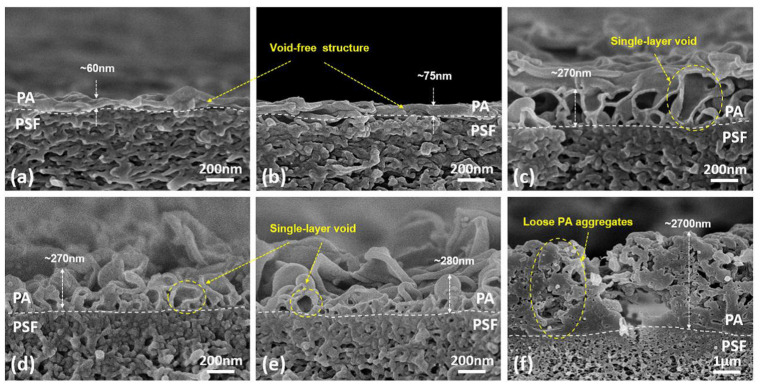
Scanning electron microscopy (SEM) images of the cross-section of the TFC membrane prepared at various MPD concentrations (TMC concentration is fixed at 0.1%). (**a**) 0.1%, (**b**) 0.2%, (**c**) 1%, (**d**) 2%, (**e**) 4%, (**f**) 20%. Reproduced from reference [60] with permission from Elsevier (2017).

**Figure 5:**
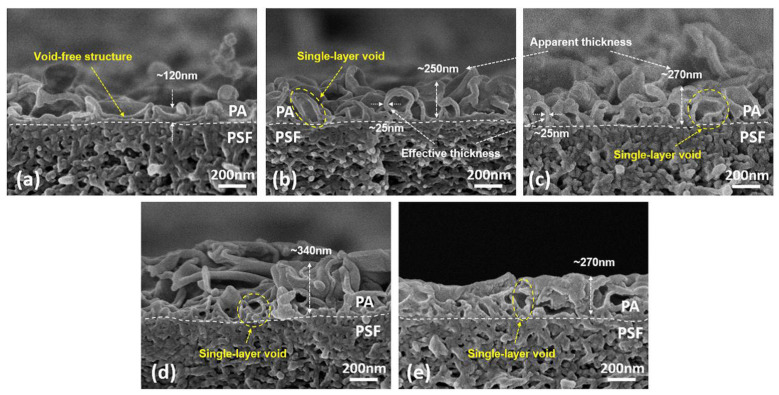
Scanning electron microscopy (SEM) images of the cross-section of the TFC membrane prepared at various TMC concentrations (MPD concentration is fixed at 2%). (**a**) 0.01%, (**b**) 0.05%, (**c**) 0.1%, (**d**) 0.2%, (**e**) 1%. Reproduced from reference [60] with permission from Elsevier (2017).

The membrane active layer controls the performance of the TFC-FO membrane regarding water permeability and salt rejection, which by increasing the TMC concentration or reducing the MPD concentration resulted in the water flux being enhanced, while the salt rejection was reduced. In which a higher MPD/TMC ratio promotes a denser PA layer via increased cross-linking, which leads to lower permeability and increased salt rejection; whereas a lower MPD/TMC ratio promotes an increase in acyl chloride content and a lower crosslinking degree of PA rejection layer, which leads to an increase in water permeability while salt rejection decreased [57,58,59]. Xu et al. [60] used PSF support layers with various MPD and TMC concentrations to build ten different TFC membranes. At low MPD concentrations of 0.1 wt.% and 0.2 wt.% and fixing TMC concentration at 0.1 wt.%, the top surface morphology of the PA layer is similar to a smooth and semi-smooth structure. It also appears very thin (its apparent thickness is less than 80 ± 7 nm) with a void-free structure in the cross-section. When the MPD concentration is increased to 1 wt.%, 2 wt.%, or 4 wt.%, the PA layer shape changes to a leaf-like structure with a rough layer and single-layer void structure. It appears thicker (ca. 2.7 μm) when MPD concentration exceeds 20 wt.% due to a large number of elongated sticks as loose aggregate on the PA surface as shown in Figure 4. While an extremely low TMC concentration of 0.01 wt.% and fixing MPD concentration at 2 wt.%, the top surface of PA shows an annular nodular structure. It also appears thin (ca. 120 nm) and void-free. When the TMC concentration is increased to 0.05 wt.%, 0.1 wt.%, and 0.2 wt.%, the morphology of the top surface of PA changes to a leaf-like structure with a rough layer and single-layer void structure. When the TMC concentration reaches 1 wt.%, the top surface seems to be a completely continuous agglomeration structure with a decrease in surface roughness as shown in Figure 5 [60]. In short, the majority of research utilized 2 wt.% and 0.1 wt.% of MPD and TMC, respectively, in the creation of the PA selective layer, demonstrating that at these concentrations the membrane can function better in the FO process. Moreover, 2 wt.% of MPD concentration might provide the formation of denser and less permeable rejection layers, as the reinforcement of solute retention tended to promote ICP reduction and hence, better membrane solute rejection, while 0.1 wt.% of TMC concentration during IP showed improvement in both water flux and salt rejection due to the dominance of reducing ICP effect over the membrane resistance [57].

Nevertheless, PA layers created during a very short IP time (e.g., 15 s) had a lot of structural defects, which resulted in the significant salt passage and, as well, decreased water permeability due to the reduced driving force across the membrane. On the other hand, the very long IP time (e.g., >45 s) resulted in a very thick selective layer, which increased the transfer resistance of both water and salt. The best IP time is 45 s for the best equilibrate between water permeation and salt rejection properties, which is given the highest water flux [61]. Whereas, the salt rejection and water flux increased with increasing curing temperature due to the rapid evaporation of the residual organic solution, which may indicate a better cross-linking of the PA layer [62]. Moreover, salt retention increases with increasing drying time of the supporting membrane saturated with amine solution before IP, and also the water permeability decreases [43]. In the future, it will be necessary to conduct more efforts be made to study the IP interaction conditions on support layers differently when applying the same IP interaction conditions to its by providing insights into the impact of properties of the support layer on the composition of the PA layer.

For past years, MPD has been employed as an aqueous monomer to manufacture TFC-FO membranes. More recently, it was found that mixing and increasing the content of polyethyleneimine (PEI) with MPD monomer in the active skin layer played a critical role in improving water permeability, salt rejection, and anti-fouling and also reduced surface roughness and skin layer thickness [63]. In another study by Xiong et al. [64], novel PAN-supported TFC-FO membranes have been explored by combining diamine monomers of N-[3-(trimethoxysilyl) propyl] ethylenediamine (NPED) and MPD as an organic-inorganic hybrid compound. With the NPED content increase from 0 to 1.5 *w*/*v*% in the PA layer, the TFC-HPAN membranes exhibit a smooth surface (19.04–9.76 nm), higher hydrophilicity (67°–42°), and higher fouling resistance as well as increased water permeability (0.437–1.439 LMH/bar) but decreased salt rejection (96.6–94.2%) because of the soft NPED/TMC segment and NPED hydrolysis. Moreover, they found that the water flux and salt flux were increased from 9.67 to 16.67 LMH and from 1.7 to 10.7 gMH using 0.5 M NaCl as DS in AL-FS orientation. In the year 2020, Nayak et al. [65] synthesized a new 4-aminophenyl sulfone (APS) monomer and used it instead of MPD to react with TMC and form the PA layer. The results show that the MPD-TMC membrane showed better rejection than the APS-TMC membrane with rejections reaching 90% and 95% for NaCl and Na_2_SO_4_, respectively. However, they found that the water flux and the salt flux did not change and remained roughly the same for both membranes.

Likewise, TMC has been employed as an organic monomer to manufacture TFC-FO membranes. In a recent study by Zhang et al. [66], mixing trimellitic anhydride chloride (TAC) monomer with a TMC solution results in a more flexible PA layer on the PSF support layer, along with increasing surface roughness, hydrophilicity, pore size, and negative charge density. This is attributed to the lower reaction of the anhydride group of TAC with amine monomer compared to the acyl chloride group of TMC. As a result, 0.04 wt% TAC membrane exhibited a high pure water permeability of 13.2 LMH/bar, salt rejection (Na_2_SO_4_: 97.6%, MgSO_4_: 92.7%, and NaCl: 34.0%), and excellent water-salt separation.

Given the above, which of the active layer and support layer has a greater role in FO performance since the effect of one of these layers on performance is closely related to the other layer. In recent years [17,67], many studies have investigated the effects of support layer properties on the formation of the active layer in order to achieve high-performance FO membranes. For example, a desired substrate surface should have water contact angles between 40° and 60°, be highly porous, have small pores, a narrow pore size distribution, and have a high pore number density. These traits reduce the ICP and S parameter. The flux stability and separation performance of the TFC FO membrane can be greatly enhanced by the lowered S parameter and ICP.

## 3. Thin Film Nanocomposite FO Membranes

With the considerable advancements in nanotechnology, the incorporation of nanoparticles into the substrate as well as into the active layer of TFC membranes is attractive because it enables changes in membrane performance without significantly altering the intrinsic membrane structure. In general, nanoparticles-modified TFC membranes in most investigations have demonstrated much higher water flux compared to unmodified TFC membranes due to increased membrane porosity, hydrophilicity, and decreased support layer tortuosity, which mitigates the ICP effect [16]. Table 1 summarizes numerous studies on nanoparticle-modified TFC membranes that have been published in the literature. The table comprises water flux and reverse salt flux with an active layer in the two different modes of AL-FS and AL-DS.

### 3.1. GO Nanoparticle

Graphene oxide (GO) is a carbon-based nanomaterial that has a single layer with a carbonous structure that is sp2-bonded. Interestingly, GO nanosheets showed a marked potential as a platform material for novel nanocomposite membrane design due to its high surface area and stronger chemical stability as well as higher hydrophilicity and excellent anti-fouling characteristics [16]. Moreover, GO has contain many functional groups such as hydroxyl (O–H), carboxyl (C–OOH), carbonyl (C=O), and epoxy (C–C) groups because of its nature of hydrophilicity [90]. Due to its many benefits, GO is compatible with many polymers and can be incorporated into polymeric membranes [91]. In the year 2015, Park et al. [68] created a PSF/GO support layer for TFC-FO membranes by integrating GO nanosheets (zero to 1.0 wt.%) into PSF substrates. It was demonstrated that GO-containing TFC membranes increased hydrophilicity and a lower structural parameter of the membrane. Upon optimal addition of 0.25 wt.% GO, the structural properties of the support layer improved, and also the formation of an effective polyamide layer. As a result, GO modified membrane exhibit higher water flux (19.77 LMH) and salt rejection (98.71%) compared with an unmodified membrane (6.08 LMH, 97.04%). However, GO loading above 0.5 wt.% caused a lower water flux due to weak GO dispersion in PSF, which resulted in the creation of a membrane with sponge-like support structures that had lower porosity and smaller pore size. In addition to the ineffective creation of a selective polyamide layer that harms the salt rejection of TFC-FO membranes. Along the same lines, Idris et al. [69] incorporated GO (in range as 0 to 1.0 wt.%) in the casting solution of a TFC FO membrane to improve osmotic power generation. At the optimal addition of 0.25 wt.% GO, the incorporation of GO not only promoted a power density of 8.36 W/m^2^ but is also able to withstand an applied pressure over 15 bar. On the other hand, the effect of different-sized GO flakes ranging from 0.01 to 1.06 μm^2^ was studied by Akther et al. [70] on the morphology and performance of the polyamide layer. They observed that the small GO flakes (MGO-8, tip sonicated for 8 h) resulted in a more uniform GO dispersion which reduced the defects of the PA layer; thus, membrane flux and selectivity improved. Whereas, the large GO flakes deteriorated the membrane performance by creating impervious regions that impeded the interaction between monomers during the interfacial polymerization process resulting in defective PA layer formation. Moreover, Saeedi-Jurkuyeh et al. [71] added GO in a selective layer and they found that these membranes can be utilized to remove heavy metals from synthetic and industrial wastewater. Pb, Cd, and Cr had the highest rejection rates of 99.9%, 99.7%, and 98.3%, respectively. Latest developments, Li et al. [92] fabricated a TFC-FO membrane for improving the water flux and anti-biofouling ability in which the substrate (TFN-S), polyamide layer (TFN-A), or both (TFN-S+A) were modified by GO. They discovered that TFN-S could greatly improve the water flux because improve the porous structure and porosity, whereas TFN-A and TFN-S+A membrane exhibited higher salt rejection and biofouling mitigation because of lower roughness and greater hydrophilicity.

### 3.2. Zeolite Nanoparticle

Zeolite is a microporous, crystalline aluminosilicate with a 3D tetrahedral framework structure and its unique features make it a material with great selectivity, high specific capacity, and exceptional resistance to chemical, biological, mechanical, or thermal stress [93]. In the year 2012, Ma et al. [72] studied incorporating zeolite NaY nanoparticles into a polyamide selective layer to enhance the performance of TFN-FO membranes. They dispersed nanoparticles in the organic solution (0.05 wt.% TMC). The addition of zeolite nanoparticles in the range of 0–0.2 wt.% changed the surface morphology, roughness, and contact angle, all of which influenced the separation properties and performance of the fabricated membranes. This addition also resulted in enhanced water flux and salt rejection at a relatively low zeolite loading level. However, a high (0.4 wt.%) zeolite loading may aid in the formation of a relatively thicker polyamide layer, which reduces water permeability and enhances salt rejection. Contrarily, the feasibility of incorporating zeolite NaY nanoparticles into a PSF-based substrate has been studied by Ma et al. [73] to improve the permeability of the polyamide layer. They found that features from zeolite loading (0.5 wt.%) improved surface porosity from 81.4% to 79.8%, reduced the contact angle from 53° to 50°, and provided additional water pathways as well as thin, sponge-like skin and a highly permeable sub-layer with straight, needle-like pores. These pores ensured a low S value and thus reducing the effect of ICP. Meanwhile, the overall thickness and contact angle of the substrate were slightly reduced with increasing zeolite loading (1 wt.%). Moreover, surface defects and unevenness with overloaded may adversely affect the integrity of the polyamide layer formed on it. Similarly, nanostructured zeolites (i.e., clinoptilolite) at a concentration of 0–0.6 wt.% were inserted into the matrix of a PES substrate and shown to be efficient in minimizing ICP effects [74].

### 3.3. TiO_2_ Nanoparticle

**Figure 6:**
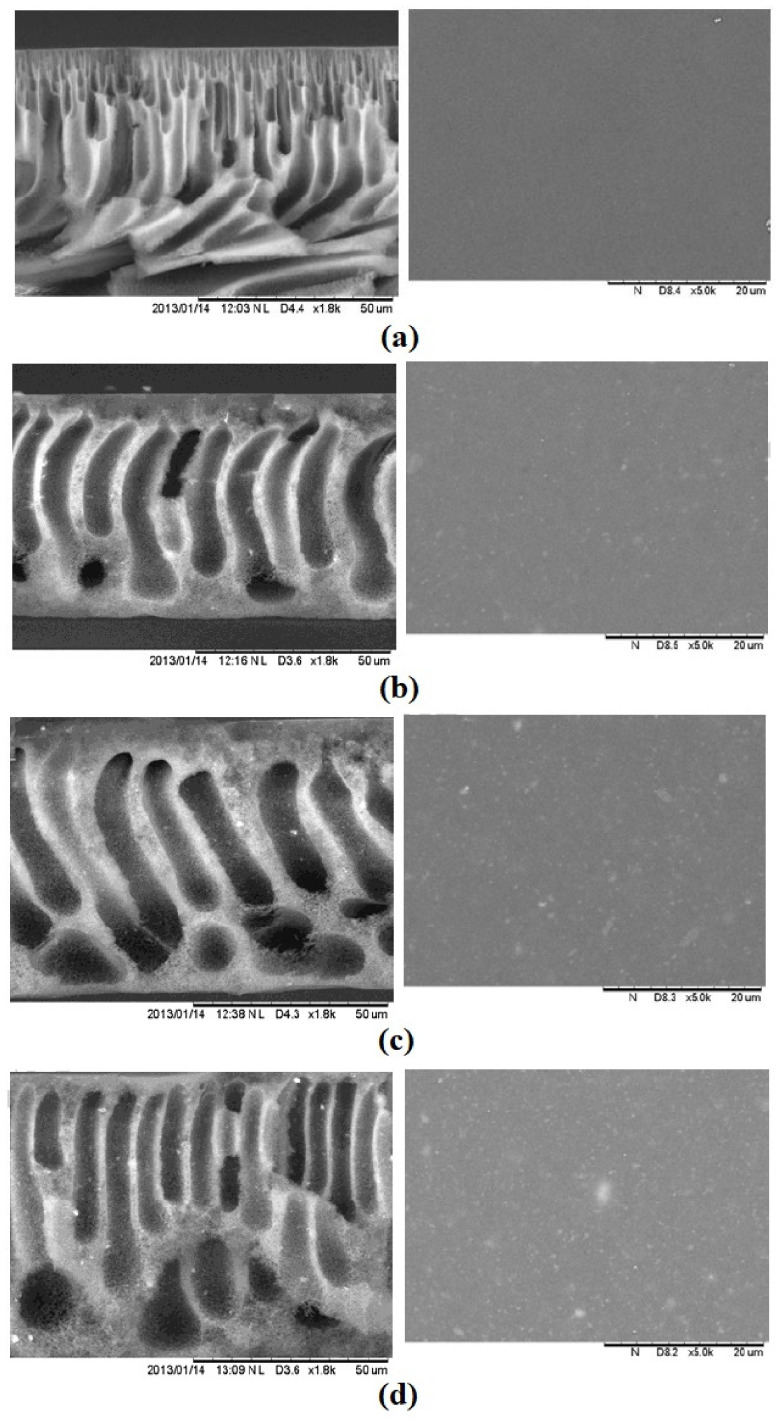
Scanning electron microscopy (SEM) images of the cross-section and top surface of PSF substrates prepared from different TiO_2_ nanoparticles loading, (**a**) PSf, (**b**) PSf 0.30, (**c**) PSf 0.60, and (**d**) PSf 0.90 substrate. Reproduced from reference [75] with permission from Elsevier (2014).

Titanium dioxide (TiO_2_) is an inorganic nanoparticle and is widely used to improve membrane hydrophilic performance since its commercially available, inexpensive, has photocatalytic behaviour, is chemically stable, has zero toxicity, and is anti-fouling [46,94]. Moreover, the TiO_2_ surface features a thin layer of water molecules, which gives it a high degree of hydrophilicity. Moreover, the photocatalytic nature of TiO_2_ aid to improve its self-cleaning ability to keep its surface clean [95]. In the year 2014, Emadzadeh et al. [75] added TiO_2_ nanoparticles, in a range of 0–0.9 wt.%, to a matrix of a PSF substrate to minimize ICP. In Figure 6, SEM pictures of the cross-sectional and top surfaces are displayed. From the figure, by adding hydrophilic TiO_2_ nanoparticles to the substrate a high number of finger-like macro-voids were generated because of delayed de-mixing during the phase inversion. Consequently, increased TiO_2_ concentration caused more nanoparticle aggregation on the substrate surface, lowering the contact area of hydroxyl groups carried by TiO_2_ nanoparticles and potentially compromising the substrate’s structural integrity. It made the membrane surface rougher and the active layer more defective. With an increase in TiO_2_ addition, water flux increased while salt rejection decreased as a result of these morphological alterations. When the TiO_2_ concentration was higher than 0.6 wt.% reverse salt flux became excessive due to due to the lower degree of cross-linking formed in the polyamide layer and nanoparticle agglomeration. As a result, the optimal amount of TiO_2_ addition was determined to be 0.6 wt.%. Whereas when it ranged from zero to 1 wt.%, the optimal value was 0.5 wt.% [76]. Additionally, Amini et al. [77] studied the chemical modification of TiO_2_ using 3-aminopropyltriethoxysilane (APTES) as a silane coupling agent and their addition to the polyamide rejection layer to avoid agglomerations on the membrane surface. Therefore, the improved water flux can be attributed to the incorporation of modified TiO_2_ nanoparticles, in the range of 0 to 0.1 wt.%, into the polyamide layer, which may be due to decreased roughness (112.48–72 nm), and increased porosity (77–81%) as well as the reduced contact angle (66.5–50.5°). Further increase in TiO_2_ concentration led to reduced solute flux. Moreover, the modification of nanoparticles increased the hydrophilic amide bonds (–NH_2_) on the surface of the membrane. Thus, the incorporation of TiO_2_ nanoparticles on the active layer allows water droplets to easily expand on it.

### 3.4. SiO_2_ Nanoparticle

Silicon oxide (SiO_2_) nanoparticles are one of the most inspirational types of inorganic nanomaterials due to their unique properties such as strong surface energy, little size, thermal resistance, nontoxic, inert nature, and fine hang in polymer solution or aqueous solution. It is also inexpensive and widely available [78,96]. In the year 2014, Niksefat et al. [79] reported on SiO_2_ incorporated into the active layer of the membrane via interfacial polymerization of MPD and TMC to enhance the membrane parameters. PSF was used for making the base layer, and SiO_2_ (i.e., in a range of 0.01–0.1 wt.%) was added to the aqueous phase (2 wt.% MPD). This study demonstrated that the improvement in water flux was caused by a decreased structural parameter (435–376 μm) which contributes to a low CP effect and thus a reduction in flux resistance, and improved membrane surface roughness (30.3–134.2 nm) and hydrophilicity (82–44°) which may be caused by the accumulation of SiO_2_ on the membrane surface. TFN membrane with 0.05 wt.% SiO_2_ provided the best water flux and salt rejection. Moreover, the overloading of silica (0.1 wt.%) may not be beneficial to FO performance and may potentially harm membrane properties. Another study has also proved that the insertion of SiO_2_ nanoparticles into the membrane support layer improved the hydrophilicity and porosity of the membrane which can effectively reduce the ICP effect. Moreover, water permeability and salt rejection were found to have improved upon the addition of SiO_2_ up to 5 wt%, but it could not enhance the FO membrane’s selectivity. It is worth mentioning that overloading of SiO_2_ content (3 and 5 wt.%) may shrink pores on the membrane surface and this did not result in serious defects on the polyamide layer. However, the water flux was found to have increased from 9.1 to 22.3 LMH and 18.2 to 41.9 LMH in AL-FS and AL-DS orientation, respectively [80]. Most recently, Islam et al. [81] incorporated super-hydrophilic SiO_2_ in both the electro-spun nylon-6 (N6) substrate and the polyamide active layer to fabricate the TFN membrane. The prepared membrane exhibited high water flux and antifouling due to 24.1 MPa tensile strength with a 14° water contact angle. In addition, the flux recoveries after fouling and cleaning operations were 98% and 95.15% for sodium alginate (SA) foulant and calcium sulfate (CaSO_4_) scalant, respectively. The developed TFN membrane’s structural stability was also enhanced by a strong contact between the selective layer and the substrate.

### 3.5. ZnO Nanoparticle

**Figure 7:**
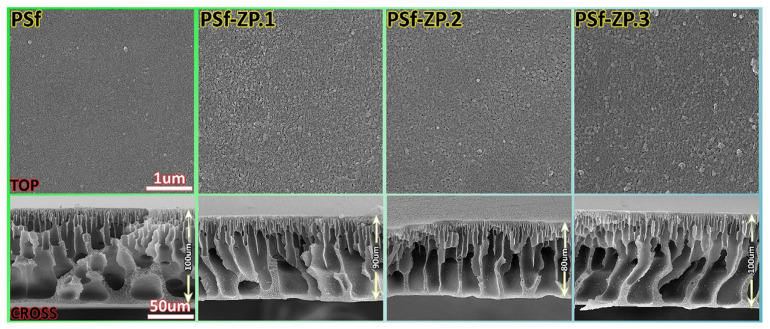
Scanning electron microscopy (SEM) pictures of the cross-section and top surface of support layers with various ZnO@PMMA loading (0, 0.125, 0.25, and 0.5 wt.%) Reproduced from reference [83] with permission from Elsevier (2022).

Zinc oxide (ZnO) has drawn increased attention since it is environmentally friendly, mechanically and chemically stable, non-toxic, and low-cost [82,97]. In addition, it is one of the best materials for creating composite membranes due to its higher surface area, increased hydrophilicity, and higher fouling resistance [98]. In the year 2018, Mansouri et al. [82] studied the influence of hydrophilic and hydrophobic modified ZnO nanoparticles incorporated in the PES matrix on FO membrane properties. Adding 0.5 wt% ZnO, the contact angle of the hydrophilic PES sublayer decreased from 56.04° to 31.57°, while it increased for the hydrophobic PES sublayer to 78.4°. Additionally, loading hydrophilic ZnO nanoparticles enhanced the pore size and porosity of the PES sublayer, while loading hydrophobic ZnO nanoparticles lowered them. Moreover, they noted that the modified PES sublayer with higher surface hydrophilicity absorbed more MPD; thus, more MPD molecules were available in the porous media to diffuse into the organic phase, resulting in a thinner PA layer with a higher degree of cross-linking due to interaction between MPD and the sublayer compared to those of hydrophobic membrane. Moreover, the TFC membranes fabricated over hydrophilic substrates revealed higher water permeability (2.66 LMH/bar) and NaCl rejection (92.12%) than those fabricated over hydrophobic substrates (1.4 LMH/bar, 89.99%). In another research study, Ghalavand et al. [83] used a poly (methyl methacrylate) (PMMA) grafted ZnO nanoparticle, in a range of 0–0.5%, to build a novel nanofiller within the PSF support layer which was coated with in situ polymerized polyamide. By the addition of ZnO@PMMA nanoparticles to the support layer forming a nano-composite sublayer, its hydrophilicity increased for the TFN membranes. In Figure 7, SEM images of the surface and cross-sectional are shown. From the figure, long finger-like morphology with a small water path length was formed as well as the overall porosity increased upon the addition of ZnO@PMMA compared to PSF due to the increase in the water transfer rate from the coagulant to the polymer film. Furthermore, all surfaces showed a smooth and flawless surface with few small pores, indicating that the addition of ZnO@PMMA helps produce a flawless PA layer. In comparison to the bare TFC-FO membrane, adding 0.25 wt.% ZnO@PMMA enhanced FO water flux and salt rejection. On the other hand, TFN membranes were made by embedding ZnO NPs (varying from 0 to 1 wt.%) into a polyamide layer by Amini et al. [98]. They discovered that all TFN membranes showed enhanced surface hydrophilicity with reduced water contact angels to 87.7° (TFN-ZnO-0.1), 79.3° (TFN-ZnO-0.2), 70.7° (TFN-ZnO-0.5), and 62.9° (TFN-ZnO-1) compared to the TFC (102.7°) due to the surface hydroxyl groups and the hydrophilic nature of ZnO NPs. In another word, including ZnO NPs in the membrane structure increases the porosity and hydrophilicity of the composite membranes, allowing them to absorb water and easily transport water molecules across the membrane. The membrane containing 0.5 wt.% ZnO is the best-performing membrane for the desalination process among the modified TFN membranes.

### 3.6. Other Nanoparticles

**Figure 8:**
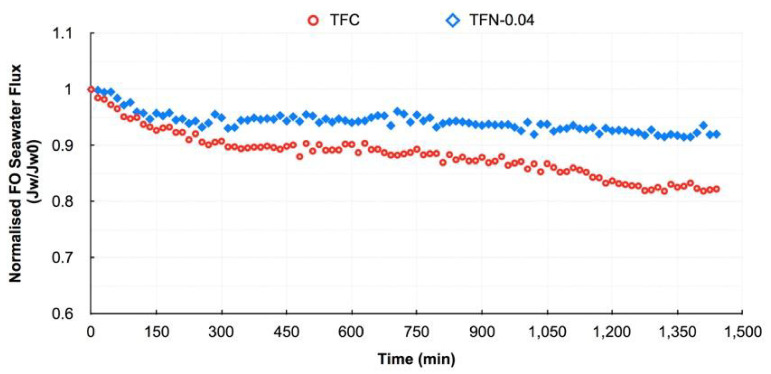
Normalized FO seawater flux decline of the membranes over 1-day (FS/Caspian seawater, DS/2 M NaCl, T/25 °C, Mode/AL-FS). Reproduced from reference [42] with permission from Elsevier (2017).

Ding et al. [84] employed hydrophilic aluminum oxide (Al_2_O_3_) nanoparticles as additives in both PSF support and polyamide layers to additionally create water channels in the substrate leading to increment in mass transfer and water permeability because they possess several advantages such as a high surface area, a large pore volume, and a high porosity. It was found that the addition of 0.5 wt.% Al_2_O_3_ NPs improved the substrate morphology, which involved high porosity (71.1%) and pore size (34.1 nm), hydrophilicity (67.7°), roughness (25.35 nm), and a finger-like structure was formed. Moreover, the structural parameter was decreased significantly from 1422 μm (pure PS substrate) to 1028 μm, which lead to lower ICP impacts. Moreover, the addition of 0.05 wt.% Al_2_O_3_ NPs to PA layer lead to higher roughness and thickness of the selective layer due to the formation of large “leaf-like” morphological structures and NPs aggregation. It was anticipated that the higher roughness, hydrophilicity, and large surface area of the active layer would result in high water flux and reduced salt diffusion. They also monitored that the modified TFN membrane demonstrated excellent FO performance and stability over long-term operation. In another study by Darabi et al. [85], ferrous ferric oxide (Fe_3_O_4_) nanoparticles were incorporated as inorganic nanofiller ranging from zero to 0.5 wt% into a PES substrate matrix because of its multiple benefits, including low toxicity, good biocompatibility, high surface area, chemical stability, and unique magnetic characteristics. The addition of 0.2 wt.% Fe_3_O_4_ to the substrate improves its main characteristics in terms of hydrophilicity (62°), porosity (87%), pore size (36.5 nm), cross-sectional morphology (longer finger-like structure), roughness (41.48 nm), and strength (3.12 MPa). This structure is preferred for FO membranes because it results in less resistance to the diffusion of water and salt and thus reduces unwanted the ICP effect of membranes. While Zirehpour et al. [42] established nano-sized metal–organic framework (MOF) particles from silver (I) and 1,3,5-benzene tricarboxylic acid. 0.04 wt.% MOF as a new category of organic/inorganic hybrid materials consisting of metal ions or clusters coordinated to organic ligands was added into the polyamide layer of membranes to improve the structure of TFC membranes for seawater desalination. This nanoparticle improved the active layer’s hydrophilicity and transport characteristics while not affecting selectivity due to the good affinity between the MOF and the PA layer. They also reported that the TFN membrane exhibited 129% higher pure water permeability in comparison with the TFC membrane as well as significantly improved performance stability throughout the testing interval. As shown in Figure 8, it was possible to see that during 1-day, the FO seawater flux declined by about 7% for the TFN membrane against about 18% for the TFC membrane which was primarily due to a decrease in driving force and fouling. It was observed from the above results that adding more or less than the optimal value of nanomaterials reduces the membrane efficiency because of the decreased membrane properties compared to the ideal membrane. In another distinction to reduce the effect of ICP, Wang et al. [99] incorporated multi-walled carbon nanotubes (MWCNTs) as potential fillers (i.e., in a range of 0–2.5 wt.%) into a PES substrate. They found that the nanocomposite substrate with 2 wt.% MWCNTs showed a desirable microstructure for promoting the separation properties of the FO process with respect to NaCl rejection rate (95%) and osmotic water flux (12 L/m^2^.h) and were higher than that of neat PES (78%, 8.2 L/m^2^.h) and commercial HTI membrane (89%, 9 L/m^2^.h), primarily due to the smoother, more open selective layer and more open interior pore structure. Moreover, the tensile moduli and strength of the substrates with MWCNTs are higher than that of the neat PES, which is useful for creating substrate supports without the need for fabric. 

### 3.7. Mixture Nanoparticles

**Figure 9:**
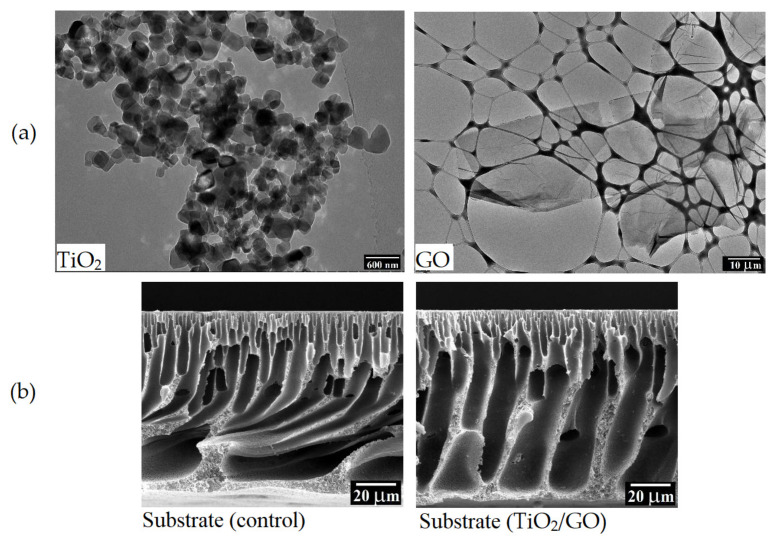
(**a**) Transmission electron microscopy (TEM) pictures of TiO_2_ and GO nanomaterial; (**b**) Scanning electron microscopy (SEM) pictures of a substrate (control) and substrate (TiO_2_/GO). Reproduced from reference [86] with permission from Elsevier (2017).

In past years, research efforts contributed to the preparation of membranes modified with a mixture of nanoparticles. Sirinupong et al. [86] incorporated titanium dioxide/graphene oxide (TiO_2_/GO) as a nanofiller into a PSF-based substrate to improve the TFC membrane performance during FO applications. As can be seen in Figure 9a, the structure of TiO_2_ differs significantly from that of the GO where TiO_2_ has a spherical shape and GO has a single flake form in nature. As a result, the TiO_2_ particles in the matrix GO-TiO_2_ material are distributed more evenly throughout the GO sheets. They reported that the presence of TiO_2_/GO in doped solution increased the viscosity from 645.3 mPa.s to 731.5 mPa.s, causing a rapid exchange of solvents–nonsolvents during the phase inversion process and thus the formation of long finger-like voids extended from the top to the bottom to facilitate water transport (Figure 9b). They also found that the inclusion of TiO_2_/GO, in comparison with the control substrate, enhanced the porosity and hydrophilicity as well as roughness of both top (from 73.11°, 12.96 nm to 68.39°, 12.64 nm) and bottom (from 69.15°, 13.06 nm to 62.88°, 17.91 nm) substrate surface. This microstructure is desirable for the FO process because it decreases the structural parameter (S) and consequently, reduces the ICP effect. Rastgar et al. [87] prepared TFN-PA membranes by introducing the ZnO-SiO_2_ core–shell nanoparticles (ZSCSNPs) into the PSF substrate as a good candidate for improving FO membrane performance. ZSCSNPs, which were more hydrophilic than ZNP, were created by first preparing ZNP using the sol-gel process and then coating them with hydrophilic SiO_2_. They found that both ZNP and ZSCSNP had the same effect but the effect of ZSCSNP was stronger. In RO tests, the NaCl rejection was almost the same after the addition of either of the two NPs due to the negligible difference in the size of the utilized NPs, i.e., 30 and 50 nm. Whereas in FO tests, the water flux of ZSCSNPs TFN membrane was higher due to the higher hydrophilicity and lower roughness of ZSCSNP than that of ZNP. Zhang et al. [88] incorporated SiO_2_/MWNTs obtained from the hydrolysis of tetraethyl orthosilicate (TEOS) on aminated multiwall carbon nanotubes (MWCNT) in a PVDF substrate to fabricate a TFN-FO membrane. Optimal membrane morphology with an appropriate pore size distribution, increase in porosity and roughness, and decrease in contact angle are all the epitome of the addition of SiO_2_@MWNTs hybrid nanomaterial. These changes finally facilitated the production of a defect-free polyamide layer. Water movement was aided by the extra mass transfer channels created by the SiO2@MWNTs in the substrate when the SiO_2_@MWNTs fraction was 0.75 wt.%. Darabi et al. [89] incorporated magnetite/zinc oxide (Fe_3_O_4_/ZnO) into both the upper and sub-layer of an FO membrane to improve its properties and performance. The inclusion of Fe_3_O_4_/ZnO resulted in a finger-like structure in the substrate and a leaf-like surface in the PA layer. Furthermore, photocatalytic Fe_3_O_4_/ZnO nanocomposite activation increased the hydrophilicity of TFN membranes under UV irradiation. Due to these morphological changes, the TFN membrane achieves a higher water flux of 78% than the TFC membrane, which also achieves the highest NaCl rejection (96.5%), and the lowest S (0.4 mm) compared to the TFC membrane (96.3%, 0.78 mm). Rastgar et al. [100] observed a 117.4% increase in FO water flux compared to the TFC membrane due to the enhanced wettability, smoother surface, and porous structure of the polyamide layer by introducing a new approach for magnetically modifying GO within the polyamide layer to create TFN-MMGO/Fe_3_O_4_ membranes. Moreover, these morphological modifications lead to reducing fouling tendency: (I) hydrophilicity, which prevents hydrophobic foulants from adhering to the membrane surface by forming a thick layer of water molecules through hydrogen bonding; (II) smoother, which reduces the area available for membrane-foulant interactions; and (III) the presence of negative carboxyl groups on the surface.

## 4. Draw Solution

**Figure 10:**
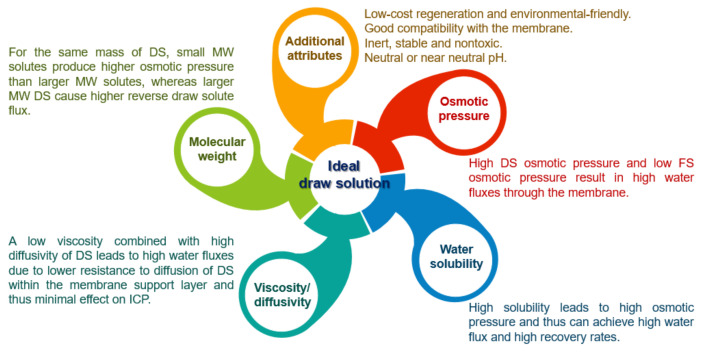
Some important characteristics of an ideal draw solution (DS).

The osmotic agent, otherwise known as the draw solution (DS), provides the driving force of the FO process that plays an important role in an FO system efficiency [101], and it is responsible for absorbing water from feed solution (FS) through a semi-permeable membrane [102]. However, there is unanimous agreement that one of the challenges facing the future development of FO is finding an appropriate draw solution capable of significantly boosting FO performance. An appropriate draw solution not only promotes the efficiency of the FO process but also saves costs of the subsequent steps in recovering and replenishing the draw solute [103]. In selection, an ideal DS must be able to meet a variety of criteria to successfully drive the FO process, including being able to: (I) generate a high enough osmotic pressure; (II) have a low viscosity to facilitate easy pumping throughout the system and improved water flux; (III) have a low reverse solute flux (RSF); (IV) be available in large quantities at a reasonable price; (v) any toxicity of the draw solute will be a big concern if there is a chance that the finished product water may get contaminated [104]. Yet, Figure 10 highlights some important characteristics of a perfect DS that could impact FO performance. In the past few years, different draw solutions have been used in the FO process, and they can be classified into two categories: electrolyte solutions and non-electrolyte solutions; and evaluated in a search for an ideal draw solute. An overview of the recent advances in various draw solutions is demonstrated in Table 2.

### 4.1. Electrolyte Draw Solutions

Electrolyte solutions make up the majority of inorganic DSs [124]. Thus, salts made up of cations (positive ions) and anions (negative ions) are the most common inorganic draw solutes. The negative ion properties of DS a remarkable role in determining the water flux, whereas reverse solute flux (RSF) is largely influenced by the positive ion properties of DS [125]. These salts can be ionized completely and generate high osmotic pressure in the aqueous solution, ensuring considerably high water flux. For instance, MgCl_2_ [26], KHCO_3_, KBr, K_2_SO_4_ [102], NaCl, NaHCO_3_ [106], KCl [107], Na_2_SO_4_ [109], MgSO_4_, Mg(NO_3_)_2_ [110], and others, have been used as DS. Inorganic salts are required to be recycled to extract pure water by the RO system for monovalent salts and the NF system for multivalent salts, and its retention of multivalent salts is higher (99%) than for monovalent salts [19]. Moreover, these salts may leach into feed water, resulting in a lot of money being spent on replenishing to keep the process running smoothly [102]. Another draw solute is NH_4_HCO_3_, which can be producing clean water after decomposition into ammonia and carbon dioxide gases using moderate heat at about 60 °C. However, ammonia might be discharged into the water due to its great solubility [105].

Compared to ions salts, Cl^–^ salts (NaCl, KCl, NH_4_Cl, CaCl_2_) showed a much larger water flux than ${\mathrm{NO}}_{3}^{-}$ salts (NaNO_3_, KNO_3_, NH_4_NO_3_, Ca(NO_3_)_2_) due to higher osmotic pressure while, Na^+^, and K^+^ salts showed the best performance as DS [108]. Moreover, divalent ions like CaCl_2_, MgCl_2_, and MgSO_4_ have a lower water flux and lower reverse penetration rate than monovalent ions like NaCl and KCl due to a larger hydrated radius (hydrated ionic radius of Ca^2+^: 0.300 nm; Mg^2+^: 0.400 nm;$\;{\mathrm{SO}}_{4}^{4-}$: 0.200 nm; Na^+^: 0.225 nm; K^+^: 0.300 nm; and Cl^–^: 0.150 nm). Moreover, because the hydrated ion of Mg^2+^ is larger than that of Ca^2+^, the retention of MgCl_2_ was higher than that of CaCl_2_ which reduces the risk of scaling [102]. Fertilizers, e.g., blended fertilizers (i.e., (NH_4_)_2_HPO_4_ and KNO_3_) [2] or (NH_4_)_2_SO_4_, NH_4_H_2_PO_4_, (NH_4_)_2_HPO_4_ [111], NaH_2_PO_4_ [112], KH_2_PO_4_ [126], are an appealing choice as DS for developing an osmotic dilution system intended for direct use in fertigation without the need for recovery and regenerating draw solute. Moreover, some studies used commercial fertilizers as DS where they were able to extract fresh water with a rate of 41% for solid fertilizer [127] and 80% for liquid fertilizer [128] from low-quality sources as feed water (e.g., wastewater). Although liquid fertilizers have a higher water dilution rate, they are less preferred than solid fertilizers because of the problems associated with storage and transportation [127]. Nevertheless, fertilizer solutions must meet the criteria for salinity water for irrigation as it affects soil fertility and thus crop productivity [111].

For the first time, Na_3_PO_4_ has been employed as DS, which delivers a high water flux (12.5 LMH) and reduces salt leakage (0.84 gMH) at pH 9. Moreover, specific RSF was lower than for a NaCl solution at 1 M due to increased complexation between Na^+^ ions and ${\mathrm{HPO}}_{4}^{2-}$, which led to the reduction in number of free Na^+^ ions. The DS regeneration was by membrane distillation (MD) using a polytetrafluoroethylene (PTFE) membrane with a 0.45 μm pore size, and it achieved high salt rejection of about 100% with a high water flux of 10.28 LMH [22].

### 4.2. Non-Electrolyte Draw Solutions

Non-electrolyte solutions are mainly organic DSs. They must be water-soluble and able to provide sufficient osmotic pressure for FO, which is crucial for achieving good water flux and recovery [129]. An organic solute with a considerable molecular size has the advantage of having minimal reverse solute diffusion. For instance, sodium alginate sulfonate (NaLS) [11], EDTA sodium salt [25], carboxyethyl amine sodium salts (CASSs) [47], sucrose [113], glucose [114], oligomeric carboxylates [115], and cobaltic complex (Na–Co–CA) [116], were able to produce a higher water flux and a much lower RSF than that with small size solutes, such as NaCl. Hydroacid complexes have been used as DS. Cu complexes (Cu-CA, Cu-MA, and Cu-TA) perform similarly to or slightly better than NaCl while, Fe complexes (Fe-CA, Fe-MA, and Fe-TA) outperform NaCl significantly in terms of water flux. As well, all complexes outperform NaCl in terms of reverse flux [117].

Polymers were also investigated as organic DS. Mostly, polyelectrolytes are an attractive option as DS, due to their good water solubility, high osmotic pressure, and large molecular size, all of which contributed to generating a high water flux and easier recovery. Moreover, the structural expansion of these solutes in an aqueous solution results in a reduction in solute leakage due to the increase in the mutual ion repulsion caused by the increase in the number of carboxyl groups in the polymer chain [10]. For instance, polyacrylic acid sodium salt (PAA-Na) [10], poly(aspartic acid sodium salt) (PAspNa) [118], polyamidoamine with terminal carboxyl groups (PAMAM-COONa) [119], poly (sodium4-styrene sulfonate) (PSS) [120], poly (isobutylene-alt-maleic acid) sodium salt (PIAM-Na) [121], and others, have been applied in FO studies, which exhibited high retention and relative low RSF. Poly (4-styrene sulfonic acid-co-maleic acid) (P(SSA-co-MA)-Na) were investigated as a potential DS. 0.25 g/mL P(SSA-co-MA)-Na exhibited a higher water flux (15 LMH) and a lower salt leakage (0.04 gMH) as compared with PAA-Na (12 LMH, 0.25 gMH) and PSS-Na (8 LMH, 0.15 gMH) because of its high osmotic pressure (32.8 bar), and large molecular size (Mw~20,000), which can be easily separated from water by NF system [31]. Although their excellent performance in the FO process and recovery system, some draw solutes are impractical due to restrictions such as commercial availability.

As well, hydrogels have recently been presented as promising draw agents in FO processes due to their ability to release water easily at a low energy cost via undergoing a reversible volume change or solution-gel phase transition in response to external stimuli like temperature [122], pressure [123], light [130], and voltage [131]. These stimuli can change the physiochemical properties of hydrogels. Razmjou et al. [131] investigated bilayer polymer hydrogels from sodium acrylate and N-isopropyl acrylamide (PSA-PNIPAM) as the first layer and PNIPAM as the second layer as FO draw materials. The first layer is responsible for water absorption from the feed while the second layer is responsible for dewatering to allow immediate release of the absorbed water at 32 °C lower critical solution temperature (LSCT). Once the dewatering layer’s water content reaches a particular level, it is possible to recover the water by increasing the temperature to LCST to induce a volume phase transition using renewable solar energy. Dewatering flux enhanced from 10 to 25 LMH when the input power of the solar concentrator increased from 0.5 to 2 kW/m^2^ [131]. Moreover, the dewatering rate in the FO process is influenced by the size of the hydrogel particles. Large hydrogel particles (500–1000 μm) recovered liquid water at higher rates under gas pressure stimulus at 6 bar, whereas smaller hydrogel particles (2–25 μm) recovered liquid water at lower rates under temperature stimulus at 60 °C [130].

However, microgels generate more water than hydrogels, which reason due to their smaller sizes, larger surface areas, and better membrane contact. Moreover, microgels are featured by negligence RSF because their sizes are larger than the pores of FO membranes [132]. Hartanto et al. [133] selected ionic thermo-responsive microgels of N-Isopropylacrylamide-co-2-(diethylamino) ethyl methacrylate (MCG-NP-DEAEMA) as a draw agent for the FO process. MCG-NP-DEAEMA showed higher water flux (45.6 LMH) and poor water recovery (44.8%) compared to non-ionic microgels containing N-isopropyl acrylamide and acrylamide (NP-AAm) (24.7 LMH, 78.7%) due to strong hydration of ionic moieties [133,134].

## 5. Effect of Operating Condition in FO 

**Figure 11:**
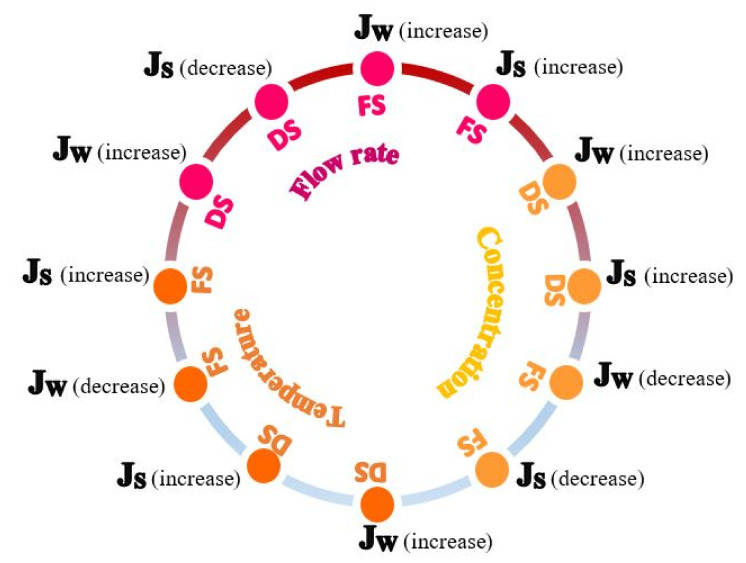
Effect increasing of operating conditions for feed solution (FS) or draw solution (DS) on FO performance.

Operating conditions have a great influence on FO performance. To make the FO process more efficient and economically feasible, the appropriate operating conditions for the FS and DS, such as flow rate, concentration, and temperature, should be determined. Table 3 provides a summary of the works that have been published in the literature on the impact of operating conditions. As well Figure 11 shows the effect increasing of operating conditions (i.e., flow rate, concentration, and temperature) for feed solution (FS) or draw solution (DS) on FO performance (water flux and reverse solute flux).

### 5.1. Flow Rate

Flow rate plays a significant role in water flux [26], reverse solute flux (RSF) [140], and membrane fouling [143] through its effect on the mass transfer mechanism. Typically, the mass transfer coefficient is enhanced with increasing flow rate, which decreases external CP (ECP) and thus improves water flux [25,144]. However, it has been observed that there is a negative effect when increasing the DS flow rate on water flux due to the increased accumulation of concentrated solute on the membrane surface, which reduces the driving force for water transfer. While the opposite occurs when the FS flow rate increases. It was most likely, due to the decrease in ECP on the feed side [145]. Some research found that a greater increase in water flux can be achieved by changing the flow rates of both rather than adjusting the flow rate of either. This is imputed to the reduced effect of concentration polarization on both sides of the membrane [136,137]. An increase in flow rate also causes an increase in energy consumption as the pump has to use more energy to force greater flow rates [146].

Flow direction in FS and DS refers to the flow pattern, which includes flow in the same direction in a co-current mode and opposite directions in a counter-current mode [125,139]. The latter is more effective because it favors a large increase in driving force and effective use of the membrane separation surface. Nonetheless, the extent of the increase is not large due to the limited size of FO membrane cells [135].

### 5.2. Concentration

Concentration is an important factor for DS as it influences reverse solute flux (RSF) [128] and water flux [147]. As an increased DS concentration will reduce the diffusion coefficient and increase the viscosity of the solution [139]. Additionally, higher DS concentration led to increases in the osmotic driving force across the membrane and thus an increase in water flux, which was attributed to an increase in osmotic pressure at the draw side [137,140]. It may also be accompanied by a slight increase in reverse solute flux (RSF) [8]. With all these, increasing DS concentration may be less effective to increase the water flux when it exceeds a certain level, it is a non-linear relationship. This is related to worsening the ICP effect/diffusion of osmotic solute in the support layer [25,116]. For example, Zou et al. [138] employed NaCl as the DS, and they found that the water flux only rose by ∼60% when DS concentration or C_ds_ was doubled from 0.5 to 1.0 M, by 35% from 1 to 2 M, and 32% from 2 to 4 M. Another study revealed that the water flux also still to be showed a slight improvement when the NaCl concentration up to 5 M [73]. Briefly, to maintain the relatively high efficacy of the FO process, it is recommended to use a DS concentration range of up to 2 M for practical purposes. 

Regarding the FS, FS concentration affects the water flux [72]. A higher FS concentration resulted in a decrease in the net osmotic-driven pressure across the membrane and consequently a decrease in water flux, which was attributed to an increase in osmotic pressure at the feed side [131]. Nevertheless, it still maintains a high rejection rate [29,116]. When FO was applied in high-salinity feed water (TDS > 20,000 mg/L), the water flux does not decline proportionally to the rise in TDS feed. It may also be followed by a slight decrease in reverse solute flux (RSF) and specific RSF. As a result, higher feed TDS plays a negative role in FO performance [139].

### 5.3. Temperature 

Temperature is regarded as an important physical parameter closely related to the physicochemical properties of FS and DS [107], and thus has a marked effect on reverse solute flux (RSF) [106], water flux [136], and membrane fouling [148]. A higher temperature will reduce the viscosity and increase the diffusion coefficient of the solution. These two factors can reduce the impact of ICP, and thus improve water flux [126]. Moreover, a temperature gradient occurs across the membrane when DS temperature rises, as it acts as an additional driving force that enhances mass transfer across the membrane [105,116]. Whereas the osmotic driving force is decreased when FS temperature rises, owing to a higher osmotic pressure at the feed side [136]. However, a further increase in temperature may lead to an increase in reverse salt flux (RSF) and consequently a decrease in water flux [141]. Some studies also found that raising the draw and feed temperatures produced almost identical water fluxes [142].

## 6. Challenges for TFC-FO

Despite the favourable characteristic features of the FO process, concentration polarization (CP), membrane fouling, and reverse solute diffusion (RSD, a major contributor) remain obstacles that hinder its effectiveness since they increase membrane resistance and lower overall membrane permeability. To improve the effectiveness of FO, there is a need to learn more about CP, fouling, and RSD, and how to mitigate them. 

### 6.1. Concentration Polarization

Concentration polarization (CP) is an inherent problem for FO processes [7]. Because most FO membranes are asymmetric, the CP generally appears on both sides of the membrane and can be further classified as internal concentration polarization (ICP) and external concentration polarization (ECP) [135.

**Figure 12:**
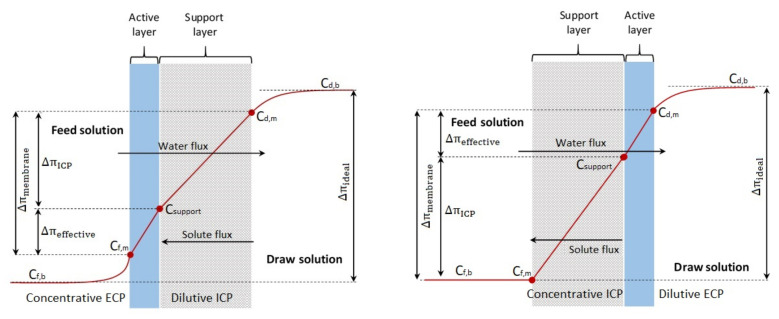
Schematic descriptions of ECP and ICP at AL–FS orientation (left-hand side figure) and AL–DS orientation (right-hand side figure).

Both ICP and ECP may occur simultaneously in FO. Generally, ECP occurs near the surface of the active dense selective layer and ICP occurs within a porous support layer, both have two types due to the absence of hydraulic pressure: concentrative CP and dilutive CP, depending on the membrane orientation. Concentrative ECP (or CECP) and dilutive ICP (or DICP) occur when the active layer is facing the feed solution (AL–FS orientation, known as FO mode), while dilutive ECP (or DECP) and concentrative ICP (or CICP) occurs when the active layer is facing the draw solution (AL–DS orientation, known as PRO mode) [144] as shown Figure 12.

#### 6.1.1. Impacts of CP

Indeed, CP can adversely affect FO performance due to the accumulation of solutes near or within the membrane surface [7]. Whether AL–FS mode or AL–DS mode, the ICP effect exhibits a more severe impact on the decrease in FO performance than the ECP effect due to the difficulty of controlling it [114]. At AL–FS orientation, dilutive ICP appears more severely on the draw side caused by the reverse solute diffusion, which provides additional transport resistance. The extra resistance lessens the water flux by dramatically lowering the effective osmotic driving force for the process [32]. Moreover, its effect can be seen more clearly on water flux by analytical and software methods as shown in Table 4. Whereas in AL–DS orientation, concentrative ICP shows less severe at the feed side due to an increase in the osmotic pressure gradient across the membrane active layer and thus an increase in water flux and solute retention [105]. However, it may show the opposite due to the presence of fouling stuck in the support layer, which reduces the porosity and mass transfer coefficient, resulting from the pore-clogging [149]. When the FO process was employed to remove boron [149], arsenic [149], tetracycline [150], and microalgae [151], ICP appeared more severe in AL–DS orientation and this contributed to poor rejection as well as a loss in water flux compared to AL–FS orientation. For this reason, the majority of researchers recommend the AL–FS orientation for FO application to prevent internal fouling and allow for less flux loss when compared to AL–DS orientation [152].

#### 6.1.2. Mitigation of CP

As an inevitable for FO, CP can be reduced and mitigated by several strategies but it cannot be eliminated. Some researchers have made an effort to fabricate and modify the membranes to decrease the impact of ICP by increasing porosity, reducing tortuosity, and improving hydrophilicity (reduced contact angle value) of the substrate through incorporating pore-forming agents such as hydrophilic polymers [54] or hydrophilic functionalized nanomaterials [82] into the membrane matrix were effective methods, which regulates the osmotic water permeation. While others employed draw solute with a high diffusion coefficient but smaller ion/molecule size and lower viscosity to produce high osmotic pressure, thus leading to a rise in the effective driving force and water flux during the FO process [103,117]. Therefore, the FO membrane is preferred to have a low structural parameter of the substrate (so S, thickness × tortuosity/porosity) to facilitate solute molecules diffusion inside the substrate, which mitigates ICP. In contrast, the adverse effect of ECP on the water flux can be minimized by changing hydrodynamic means such as an increased flow rate or turbulence since water flux increases with cross-flow velocity increases [135,147]. Adding to this, optimizing the water flux is an effective approach to minimizing ECP [89]. However, ECP is comparatively negligible when pure water is used as a feed solution; but it appears important under special conditions such as non-pure water and a low flow rate.

### 6.2. Membrane Fouling

**Figure 13:**
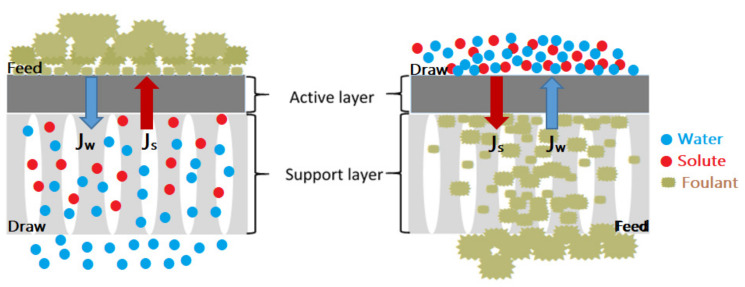
Schematic descriptions of membrane fouling at AL–FS orientation (left-hand side figure) and AL–DS orientation (right-hand side figure).

Like concentration polarization, membrane fouling is an inherent drawback for FO processes [129]. Its occurrence depends on a decrease in mass transfer (i.e., declined water penetration) which negatively affects the efficiency and lifetime of the membrane [101]. In Figure 13, membrane fouling in FO can be divided into external and internal fouling, it depends on the orientation of the membrane. At AL–FS orientation, fouling occurs on the active layer surface through the adhesion and adsorption of foulants, which enhanced cake/gel layer formation and causes external fouling. Whereas in AL–DS orientation, fouling occurs on or inside the support layer based on foulants size. If it is smaller than the pore size, it leads to pore clogging of the membrane or internal fouling, which enhances the hydraulic resistance and ICP effect. However, if it is larger, it leads to external fouling. Moreover, internal and external fouling may occur simultaneously when the feed solution includes different sizes of foulants [154]. To prevent pore blockage and a severe ICP effect, it is advised to employ the AL–FS orientation rather than the AL–DS orientation for the FO process [148,149].

Membrane fouling is based on the foulant’s nature, and there is typically categorized into four groups including organic fouling, inorganic fouling/ scaling, colloidal fouling, and biofouling/microbial fouling. Organic fouling is caused by macromolecular organic compounds such as protein, alginate, humic acid (HA), and bovine serum albumin (BSA); inorganic fouling is caused by crystallization/scaling of a sparingly soluble salt such as calcium carbonate, calcium sulfate, barium sulfate, magnesium salts, and silica; colloidal fouling is caused by the deposition of colloidal particles such as silica; and biofouling is caused by adhesion/deposition of bacteria with growth to forming a biofilm [7,154]. Apart from individual fouling, interactions between different types of foulants also play an important role in membrane fouling, such as organic (sodium alginate, bovine serum albumin)–inorganic (silica nanoparticles) foulant [155], organic (alginate)–colloidal (silica) foulant [156], microorganisms–organic foulants (protein, polysaccharide)–inorganic elements (ammonia nitrogen, phosphate) [157], and organic foulants (protein, polysaccharide)–inorganic elements (sulfate, calcium, magnesium, and silicon)–biological [158]. These foulants interact with the membrane surface exacerbating the membrane fouling.

**Figure 14:**
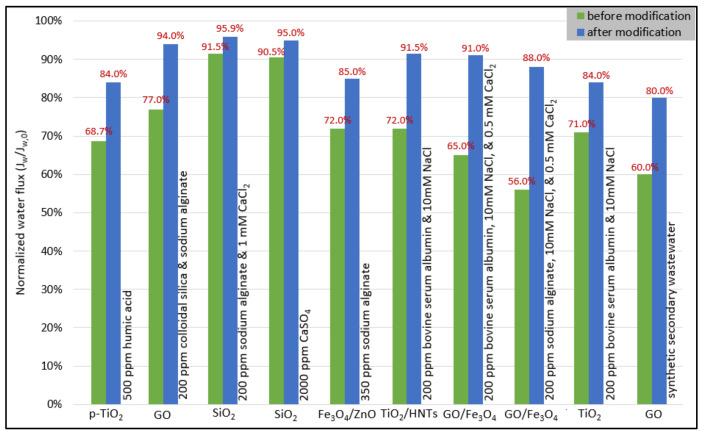
The effect of p-TiO_2_ [46], GO [69], SiO_2_ [81], Fe_3_O_4_/ZnO [89], TiO_2_/HNTs [94], GO/Fe_3_O_4_ [100], TiO_2_ [161], and GO [162] on various foulants of the composite membrane in FO mode.

To alleviate fouling, improving membrane surface properties contributes significantly to reducing membrane fouling. It has been reported that surface morphology is the most important factor influencing membrane properties, its specific roles in membrane fouling are still unknown [159]. The roughness of the polymeric membrane is an inherent feature of composite membranes. For example, Elimelech et al. [160] found that surface roughness increases result in increases in the additional attachment to the membrane surface (when especially in comparison to the ideal case of a smooth membrane) and thus more severe fouling. Many researchers have pointed out the importance of surface roughness in increasing the attraction rate between particles or between the addition and a surface. These studies are directly related to our study of rough composite polymeric membrane fouling [160]. Moreover, Li et al. [159] investigated the effects of alginate adhesion fouling on surface morphology roughness. The adhesion of an alginate chain was proposed to occur via two main paths: fitting adhesion and direct adhesion. Alginate chain bending and adhesion were found to be endothermic and exothermic processes, respectively, based on thermodynamic analyses [159]. Moreover, introducing hydrophilic nanoparticles to membrane technology decrease their contact angle and tends to increase their hydrophilicity, improving pollutant separation performance. For example, GO-modified TFN membranes (MGO-0 and MGO-8) feature hydrophilicity, smoothness, and surface negativity as well as negatively charged functional groups, which have enhanced anti-fouling and selectivity by repulsion of the negatively charged foulants and salt ions [72]. It can reduce the adsorption of large bovine serum albumin molecules on the surface of the polyamide selective layer by improving the hydrophilicity of the TFC membrane by adding a TiO_2_/HNTs [80] and TiO_2_ [161] to a substrate of the TFC membrane. All the studies mentioned in Figure 14 modify the TFC membranes surface by introducing nanomaterials to improve the performance of FO and resistance to various foulants. On the other hand, hydrophilic sulfonated polymers can also be employed to modify membranes as they provide better performance for TFC-FO membranes with resistance fouling properties [55,56]. Interestingly, high water permeability can be detrimental to a membrane’s ability to anti-fouling because all solutes, including fouled macromolecules, struggle to get through the membrane pores [59,99].

**Figure 15:**
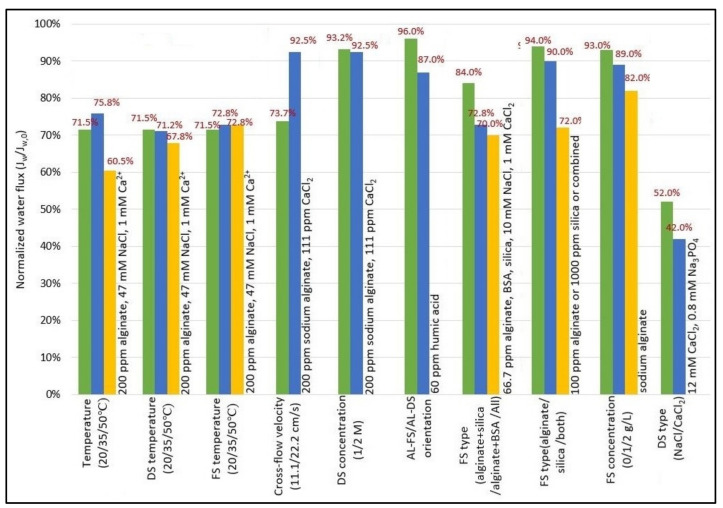
The effect of temperature [142], cross-flow velocity [143], DS concentration [143], membrane orientation [148], FS type [155,156], FS concentration [163], and DS type [164] on membrane fouling.

In the FO process, the operation conditions influence membrane fouling through shear force and drag force. Higher flow velocity helps to reduce membrane fouling by enhancing the shear force to reduce foulants’ ability to deposit on the membrane surface [143,155]. Meanwhile, higher temperature [142] and DS concentration [143] lead to worsening membrane fouling by increasing the drag force to encourage foulant deposition. Moreover, membrane fouling is considered a function of feed concentration. High organic foulants concentration in FS led to more severe membrane fouling due to the cake layer thickness increase [163]. Moreover, FS and DS types affect membrane fouling. Regarding the DS, the passage of scaling precursors such as${\;\mathrm{Ca}}^{2+},{\;\mathrm{SO}}_{4}^{2-}$, ${\mathrm{PO}}_{4}^{2-},\;$and${\;\mathrm{CO}}_{3}^{2-}$, present in DS through the membrane resulted in the formation of a cake layer by their interaction with foulants in the feed side, thus, aggravating severe scaling [164]. Figure 15 shows the effect of FS and DS types as well as operating conditions on membrane fouling.

Several of the latest FO research studies have highlighted the importance of feed spacer design in reducing fouling and concentration polarization [165]. A feed spacer is an essential component in spiral wound membrane (SWM) modules, generally in the shape of a diamond net that includes a dual-layer structure of filaments attempting to cross each other. Feed spacers, not just separate membrane leaves, thereby producing feed channels, but also act as a “turbulence promotor”, enhancing flow mixing, mass transfer, and minimizing CP. Moreover, the inclusion of a feed spacer introduces two major drawbacks. The feed channel pressure (FCP) decreases (pressure drop from channel inlet to outlet) within the cross-flow channel caused by feed spacer resistance to the fluid flow. The other one is stagnant zones, which usually form at the intersections of spacer filaments and the contact surface between the feed spacer as well as membrane, where flow tends to slow and foulant deposits, exacerbating the CP phenomenon and causing fouling [166]. Ali et al. [165] also found the dynamic feed spacer used to reduce the fouling in the FO membrane process by using 3D printed dynamic turbo-spacer to reduce fouling in a FO process for osmotic seawater samples were diluted. Due to its exceptional hydrodynamic behavior, where the turbo-final spacer’s foulants resistance has been more than 2.5 times less than the reference spacer after six separation cycles (1 day/cycle).

### 6.3. Reverse Solute Diffusion

Reverse solute diffusion (RSD) is the penetration or diffusion of a solute through a membrane during the FO process from the draw side to the feed side due to the solute concentration gradient. This movement seriously reduces the performance of the membrane by accelerating CP and reducing the osmotic driving force (i.e., declined water flux) [8,103]. Nevertheless, a high RSD contributes to a significant loss of draw solute toward the feed water and thus contamination of the feed water. For example, some multivalent ions, such as Ca^2+^ and Mg^2+^, may interfere with foulants on the feed side during reverse diffusion, promoting organic fouling growth [167]. Moreover, foulants collecting on the membrane surface can enhance solute leakage by increasing osmotic concentration near the membrane surface between the cake layer and the active layer [50]. Therefore, the contamination risk must be assessed when selecting/designing both FO membranes and drawing solutes for a FO application.

As a unique feature of FO, RSD has been utilized for pH adjustment /or enhancement of anti-scaling resistance by including an anti-scaling agent, such as H^+^ and EDTA, in draw solution chemistry, which can be caused to sequester Ca^2+^ in feed solution during reverse diffusion and thus reduce calcium phosphate scaling [164]. Moreover, RSD had a beneficial effect on sodium alginate (SA) through the interaction between SA and permeable Ca^2+^ from the draw side leading to the formation of calcium alginate (Ca-Alg) on the membrane surface on the feed side, which can be used as a recycled material. It is interesting to note that water flux rose dramatically when CaCl_2_ concentration increased, whereas SA concentration had little effect on water flux in FO [168]. An overview of recent studies on factors influencing RSD is demonstrated in Table 5.

To mitigate RSD, it is preferred to enhance membrane selectivity (so lower B/A ratio, higher J_w_, and lower J_s_) by the development of a FO membrane to have a top thin polyamide (PA) rejection layer and porous membrane support, which provides higher water permeability [72]. Furthermore, using multivalent ions with low diffusion coefficients reduces solute diffusion due to their large ionic sizes but this may lead to a more severe ICP and increased fouling risk [103,129]. In recent years, employing polyelectrolytes-based draw solutions to reduce solute diffusion more effectively than with conventional solutes like NaCl, due to their structural expansion in an aqueous solution. However, they did not address diffusion coefficients and ICP effects [10]. However, more work is needed to understand how to control and reduce RSD without side effects in future studies.

## 7. Energy Consumption in FO Compared to RO

As a quantitative measure of energy consumption per volume of produced water, specific energy consumption (SEC) is the best indicator to determine how energy-efficient of a membrane process [167]. FO is gaining popularity as a process that consumes less energy compared to other processes as it derives its energy from the draw solute. This may be one of the most attractive features of the FO system, especially during energy crises. It should be noted that DS concentration [24], temperature [126], FS type [128], and flow rate [146] are the most important operating factors that affect the energy consumption of the FO system. For example, SEC was significantly reduced when FS was changed. SEC of the secondary pulp and paper industrial effluent was 0.25 kWh/m^3^, significantly higher than 0.11 kWh/m^3^ with a humic acid aqueous solution of 5 mg/L and 0.09 kWh/m^3^ with distilled water at 1 L/min and 2 M urea as DS [32]. Moreover, primary wastewater causes severe fouling of the FO membrane which leads to reduced water flux and thus higher SCE of 0.17 kWh/m^3^ [128]. On other hand, an osmotic pressure gradient across the membrane is low at a lower draw concentration (22 g/L multicomponent fertilizer), requiring more circulation to achieve reasonable water flux and thus an increase in SEC (0.060 kWh/m^3^). Whereas an osmotic pressure gradient is good at higher draw concentration (200 g/L), which results in acceptable water flux and lower SEC (0.036 kWh/m^3^). Although a higher concentration leads to a significant reduction in energy consumption and an increase in water flux, RSF is still high and more dilution is needed to get it down to a level where it may be utilized safely in agricultural activities [112]. The flow rate decrease from 100 to 10 mL/min did not significantly reduce the water flux but did significantly reduce the energy use from 1.86 to 0.02 kW/m^3^ at 1M fertilizer as DS [127]. A higher temperature has a reverse impact on SEC [126]. As a result, operating the system at a low flow rate, high temperature, high draw concentration and low feed concentration with low energy consumption is more energy efficient.

When compared to RO, the techno-economic analysis has shown that FO and RO can be combined to consume less energy overall. It demonstrates that when compared to a standalone RO system, the hybrid FO-RO system might cut energy usage from 1.95 to 1.47 kW h/m^3^ [174]. In another study by Yangali-Quintanilla et al. [152], an FO-RO system that dilutes seawater by collecting water from secondary wastewater effluent requires roughly 1.3–1.5 kWh/m^3^. This uses less energy than standalone single-pass RO, which typically uses roughly 2.5 kWh/m^3^ of energy. Interestingly, FO is utilized to pre-treat highly scaling saline before RO. Therefore, RO is shielded from frequent scaling and cleaning. It will also be more energy efficient than the standalone RO process after operating for 60 min [175]. Accordingly, Seo et al. [176] developed a numerical model to analyze the specific energy consumption (SEC) of a hybrid FO-RO process compared to a stand-alone RO process. At 30, 40, and 50 bar of RO applied pressure, the RO SEC for the FO-RO hybrid process considering FO energy consumption is 2.68, 0.31, and 0.15 kWh/m^3^ less than the stand-alone RO process.

## 8. Other FO Applications

In the past few years, many studies on FO have been conducted, efforts have been devoted to developing high-performance FO membranes and suitable draw solutes with reasonable regeneration technology [11]. To enhance the practical applications of FO, it is necessary to search for applications in the field of agriculture such as for fertigation or fertilized irrigation as well as look for the potential industrial applications, that do not need an external supply of DS or a recovery procedure for diluted DS.

### 8.1. From an Agricultural Perspective

From this perspective, studies on potential direct fertigation applications for the FO process utilizing fertilizers have been conducted to investigate the process sustainability in agriculture to feed an irrigation system. Fertigation is an agricultural irrigation procedure in which water-soluble fertilizers are added to irrigation water [111]. Thus, the idea of recovering water from feed water resources is to thin out a fertilizer solution that can subsequently be used to fertigation farmland as well as improve and boost crop output on a large scale [146]. Fertilizer drawn-forward osmosis (FDFO) has been proposed as a potential method for lowering the quantities of potable and desalinated water utilized in the process. The most intriguing conclusion from this study was that the diluted draw solution may be used straight for irrigation without the need step for separation [111]. Although this process has several advantages, it has limitations that will affect the economic and technical feasibility of FDFO applications, namely the loss of the main fertilizer components as it moves from DS to FS, which also affects the rate of dilution that can be achieved for fertilizers [2]. In short, all the constraints faced by this process must be taken into account to find solutions to extract the most water from the feed water.

In recent studies, fertilizers with different osmotic pressures were selected as DS to achieve the purpose of high-quality water recovery from synthetic wastewater (containing microplastics and nanoplastics) [1], domestic wastewater [128], local municipal wastewater [128], synthetic brackish water [146], and raw sewage [177]. However, most of the reported water flux was relatively low using CTA membrane due to solute build-up in the final FS which was mainly attributed to reverse nutrient ($K^{+}$, ${\mathrm{NH}}_{4}^{+}$, and ${\mathrm{PO}}_{4}^{3-}$) fluxes. This permeation was inversely related to the hydrated solute radii:$\;{\mathrm{NH}}_{4}^{+}\left(0.25\;\mathrm{nm}\right)<K^{+}\;\left(0.34\;\mathrm{nm}\right)<{\mathrm{PO}}_{4}^{3-}\;\left(0.49\;\mathrm{nm}\right)$. Moreover, the steric hindrance (size exclusion) plays an important role in this transition. The reverse phosphate flux was one order lower than that of ammonium and potassium due to the relatively larger wet radius and stronger electrostatic repulsion. Much less phosphate seepage is beneficial for nutrient uptake by Greenwall plants [177]. It is noteworthy that a fertilizer blend can be used with lignin salt (e.g., sodium lignin sulfonate, NaLS) to increase osmotic pressures and improve plant development conditions [11].

Interestingly, pressure-assisted forward osmosis (PAFO) has been employed rather than FO to improve the rate of water production. Potentially, PAFO eliminates the need for further posttreatment. The additional water flux generated throughout the process can increase the final DS fertilizer dilution beyond osmotic equilibrium, making the finished water suitable for direct fertigation [178]. Furthermore, a low concentration of DS (0.25 M KCl) was used, allowing diluted KCl to be also used immediately for fertigation after PAFO operation [179].

### 8.2. From an Industrial Perspective

From this perspective, only a few studies have been carried out in recent years to seek potential industrial applications for the FO process to examine the sustainability and stability of the process in the long term as well as to assess the membrane’s durability. For instance, Guo et al. [180] reported preparing a cationic membrane grafted with IL-NH_2_ which was used to water recovery from Safranin O dye (100 ppm) during long-term FO operation. They found that water flux maintained 90% of the initial water flux during longer operation durations (up to 10 h). Moreover, the water flux had been fully recovered and was only a slow loss after 15 min of physical washing. Dong et al. [181] reported about preparing a Fe^3+^-bridged membrane that features good water permeability and high pharmaceutical retention can efficiently reclaim pharmaceuticals such as trimethoprim (TMP) and sulfamethoxazole (SMX) from their dilute solutions. After 10 h of continuous operation, water permeation reduces by 12% with 50 ppm TMP as the FS and 2 M MgCl_2_ as the DS. After 30 min of cleaning, water flux restores to 98% of its original value after 10 h experiments. Arjmandi et al. [182] reported about preparing a novel integral thin-film-based porous matrix membrane (TF-PMM) that can benefit orange juice concentration removal. They discovered that water flux maintained at 80% of the initial water flux during 30 h from an operation. Moreover, industrial wastewater with different heavy metal ions was selected as the feed water to realize the purpose of water recovery using a novel FO membrane (PA layer on Cu-alginate hydrogel intermediate layer-modified PES support) [183], a novel nanoporous thin film inorganic (TFI) membrane (made by sol-gel process driven by tetraethylorthosilicate) [184], and TFN membrane based on a zwitterion-functionalized metal–organic framework (MOF) [185].

As seen above, membrane durability is one of the major issues related to the effective commercialization of FO applications. The ability of the membrane to resist destruction and permanent change in performance over time was improved by modifying the membrane structure to achieve stability in the permeability of water and dissolved solutes/foulants rejection while maintaining the mechanical force of the membrane. It is worth mentioning that the protocol for the long-term durability of the FO membrane was conducted by running the experiments for 10 days using wastewater secondary effluent (SE) and NaCl as FS and DS, respectively. SE was collected from Blue Plains Advanced Wastewater Treatment Plant (AWTP) in Washington, USA. The membrane durability demonstrated excellent performance in contaminant rejection potential while did not deteriorate the membrane with time [186]. 

## 9. Case Studies

To know the real state of the FO process, FO offers a workable remedy for real wastewater treatment to avoid the possible hazards of wastewater management using popular disposal methods and treatment processes. However, demonstrating its performance in field reality will be a necessary future step for the successful implementation of the technology. Several researchers in different countries have presented case studies about the use of FO technology in treating real feed water but on a pilot plant scale.

### 9.1. Pilot Plant A

In Qatar, the osmotic concentration (OC) process (adapted from the FO process) is employed as a “one-pass” to remove water from real process water (TDS: 2000 mg/L) generated from a natural gas processing facility in gas fields. Arabian Gulf seawater (TDS: 40,000 mg/L) is applied as DS due to the gas field’s proximity to the Arabian Gulf. In OC, pilot-testing results revealed that the TFC hollow fiber Module (9.9 L/m^2^h) had a larger flux than the CTA hollow fiber Module (1.7 L/m^2^h) during a 50-h of continuous operation, as well as a lower reverse solute flux for the majority of the ions. After water extraction of 75%, diluted DS is discharged directly into the Arabian Gulf while concentrated FS is injected into a gas well [187].

### 9.2. Pilot Plant B

In Kuwait, a polyelectrolyte-driven FO process is utilized for desalinating beach well seawater as a sample of the Arabian Gulf seawater (AGS). AGS was obtained from a beach well located at Desalination Research Plant (DRP) in Doha, Kuwait. It was introduced that FO desalination as a ‘‘single-stage’’ may produce product water with excellent quality. After 30 days of continuous operation, the 10 m^3^/day capacity FO pilot plant’s performance remained stable, and the TDS of the product water was within 100 to 150 mg/L from its initial TDS (for Arabian Gulf seawater) of 35,801 mg/L across hollow fiber module (TOYOBO), which was a water recovery ratio of about 30% with a discharge of brine with the TDS of 49,518 mg/L. Interestingly, diluted DS can regenerate by the low energy system such as waste heat [188].

### 9.3. Pilot Plant C

In Spain, a fertilizer-driven FO (known as FDFO) process was used for municipal wastewater reuse in direct fertigation (i.e., injection of fertilizer into an irrigation system). The feed was an effluent of membrane bioreactor (MBR) from a wastewater treatment facility at San Pedro del Pinatar in Murcia, Spain. The 3 m^3^/h capacity FO pilot plant used six TFC flat-sheet modules (Porifera) of 84 m^2^ total area, fed with a 2 m^3^/h DS (MgCl2) flow. Over the 480 days of plant operation, the FO pilot plant can achieve a stable permeate with high quality for wastewater treatment and reuse in the long term. Four NF membranes module (Filmtec) having a surface area of 60.8 m^2^ were employed to provide the final product fertilizer solution for irrigation [189].

### 9.4. Pilot Plant D

In Australia, a pilot-scale FDFO-NF system is utilized for desalinating saline groundwater (TDS: 2491 mg/L) produced during coal mining activities at one of the coal mining sites in New South Wales, Australia, and reuse in agriculture to feed an irrigation system. This process employed two CTA-FO membrane modules (HTI) as the desalination step and a TFC-NF membrane module (Woongjin Chemicals) to treat the diluted DS ((NH_4_)_2_SO_4_) to meet the irrigation quality standards. The pilot plant was implemented with a capacity of 1000–4000 L/day for six months and was the capacity to produce nutrient-rich irrigation water to support direct fertilization [190].

### 9.5. Pilot Plant E

In South Korea, the FO-RO process operating in osmotic dilution (OD) mode is applied to treating real wastewater secondary effluent (SE) with seawater (SW) desalination. SE (for FS) was collected from wastewater at a coal-fired power plant, and SW (for DS) was collected from the East Sea. This process employed four PA-FO membrane modules (Porifera) as the treatment SE step and three RO membrane modules (Dow Filmtec) to desalinate diluted SW. During the plant’s five-month operation, the FO-RO pilot plant (capacity of 21.8 m^3^/day) can achieve sustainability due to its simple fouling control, low energy requirement, and superior ultimate water quality. The diluted SW was used as cooling water in the power plant [191]. 

In addition to what has been mentioned and what will be mentioned in future studies, the FO technology achieves sustainability in saving energy, seawater desalination, and treating real pollutants resulting from factories and plants, as well as in providing water suitable for potable use. Moreover, FO might be suitable to treat two wastewaters in only one step. In Oman, the 100 m^3^/h capacity of the FO plant is installed at Al Khaluf near the Arabian Sea by Modern Water Company and used for Arabian seawater desalination for drinking water purposes. After 35% water recovery to dilute the DS by FO, the dilute DS is desalinated by RO to produce potable water (TDS: 120 mg/L) [192]. Within the next decade, I anticipate that the technology will take off into the market as more and more companies begin to incorporate it into their water purification systems. It will be able to sanitize water for industrialized nations in addition to significantly assisting developing nations who lack the financial means to pay the astronomical energy costs associated with desalination facilities.

## 10. FO Future

Since 1960, the idea of using forward osmosis technology to create clean water has been floated. This technology has advanced tremendously over time and is without a doubt one of the most promising technologies used in applications variety of municipal, industry, irrigation, and desalination. It has many benefits, but it still faces many challenges that must be overcome in future studies, where a strategy must be considered to develop (I) high-performance and long-term membranes to mitigate concentration polarization and fouling, and (II) a strong draw solution to features high water flux, low back flux, and easy recovery. Furthermore, another significant challenge is the transition of FO research from the lab-scale to large-scale implementation, which is usually challenging and requires collaboration between membrane scientists, engineers, and end-users.

Now with FO limitations requiring the replacement of membranes after a certain amount of time, we are pleased to provide future solutions to these limitations and thus become new research directions. To create FO membranes that are more effective, reliable, and affordable, future research should concentrate on: (I) scaling up the manufacture of eco-friendly membranes, (II) lowering the cost of the membrane materials by utilizing material residue (e.g., eggshells [193]) and green solvents (e.g., dihydrolevoglucosenone [194], ionic liquids [195]), and (III) adding novel sustainable nanomaterials in a substrate and/or the thin selective layer (e.g., carbon quantum dots [196], graphene quantum dots [197]). Although there is little information on the conversion of FO membranes into sustainable membranes, future development of FO membranes should take into account these sustainable materials.

Moreover, FO still needs to design a sustainable draw solution that achieves equilibrium between osmotic pressure and reverse solute flux (e.g., magnetic nanoparticles [198]). A regeneration system is also being designed for a draw solution that adopts the use of sustainable and renewable energy (e.g., a renewable-powered membrane distillation system [199]). Even if it sounds economical and technical, this still needs extensive research.

## 11. Conclusions and Prospects

FO process shows great potential in applications such as municipal wastewater treatment, industrial wastewater treatment, and removal of various solutes, which have gained more attention in recent years. However, FO is still having trouble finding a draw solution that offers low solute flux, high water flux, and simple regeneration as well as a suitable membrane that offers a support layer with high flux and an active layer with high water permeability and solute rejection from both the feed solution and the draw solution at the same time. This work reviews the following aspect:

Membrane surface characteristics and performance, such as pore structure, hydrophilicity, surface roughness, and water flux, are closely related to membrane fabrication. This article provides a perspective on the role of the active layer and substrate in the performance of FO and also advances in the modification of FO membranes utilizing nanoparticles (NPs) such as graphene oxide (GO), zeolite (NaY), titanium dioxide (TiO_2_), silicon oxide (SiO_2_), zinc oxide (ZnO), and mixture NPs. The functions and effects of NPs were evaluated when combined with the membrane to enhance selectivity, permeability, internal concentration polarization (ICP), fouling, and stability.

The choice of an appropriate draw solution is crucial for the economical and energy-efficient operation of FO. A perfect draw solution would have a high water flux, a low reverse solute flux (RSF), be nontoxic natural, and be simple to regenerate. The draw solution has been classified into electrolytic solutions and non-electrolytic solutions. We would like to help the main categories of draw solutions discussed in this review develop further efforts to create effective draw solutions.

Flow rate, concentration, and temperature of feed solution and draw solution are the most important operating conditions that affect the FO process. The optimum operating condition is usually determined by either the maximum recovery rate or minimum final draw solution concentration.

Concentration polarization (CP), membrane fouling, and reverse solute diffusion (RSD) are issues with the FO process. RSD may be decreased and membrane fouling may worsen due to severe CP.

Moreover, we highlight factors that influence the energy consumption of the FO process and compared them with the reverse osmosis (RO) process. Regarding RO SEC and recovery rate, the FO-RO hybrid process outperforms the standalone RO process. In addition, applications were identified in the FO process, particularly in the field of agriculture such as for fertigation or fertilized irrigation, due to the significant water consumption in this field, and also know the potential industrial applications. Finally, case studies and future studies ideas in the FO process were discussed.

## Figures and Tables

**Table 1:** Summary of the studies on FO performance of nanoparticle-modified TFC membranes.

Support Layer Polyamide Active Layer Nanomaterial	Experimental Operating Conditions				FO Performance				Intrinsic Properties	Reference
	Feed Solution	Draw Solution	Temp.	Flow Rate	FS–AL (FO Mode)		DS–AL (PRO Mode)			
					Jw; LMH	Js; gMH	Jw; LMH	Js; gMH		
14% PSF/1% PVP 2% MPD/0.1% TMC 0.04% MOF in organic solution	DI water	2M NaCl	25 °C	21 cm/s	46	102.3	—	—	A = 4.7 LMH/bar B = 0.6 LMH S = 238 μm	[42]
16% PSF 2% MPD/0.15% TMC 0.25% GO in dope solution	DI water	0.5M NaCl	25 °C	1.8 L/min	19.77	3.4	40.5	6.5	A = 1.76 LMH/bar B = 0.19 LMH R (NaCl) = 98.71% S = 191 μm	[68]
18% PSF 2% MPD/0.1% TMC 0.25% GO in dope solution	DI water	1M NaCl	24 °C	1.5 L/min	14.65	3.62	30.95	6.6	A = 1.91 LMH/bar B = 0.24 LMH R (NaCl) = 98.67% S = 726 μm	[69]
12% PSF 4% MPD/0.1% TMC 0.01% GO-8h in aqueous solution	DI water	0.5M NaCl	22 °C	12.6 cm/s	24.7	5.19	41.9	8	A = 3.71 LMH/bar B = 0.89 LMH	[70]
16% PSF/4% PEG-400 2% MPD/0.1% TMC 0.008% GO in aqueous solution	DI water	2M NaCl	—	12 L/h	34.3	1.1	—	—	B = 3.9 LMH/bar A = 1.1 LMH R (NaCl) = 96.7% S = 119 μm	[71]
15.5% PSF/0.5% PVP/3% LiCl 1% MPD/0.05% TMC 0.02% zeolite in organic solution	DI water	1M NaCl	—	0.5 L/min	13.8	7.08	28.8	13.76	A = 5.27 × 10^−12^ m/s.Pa B = 15.1 × 10^−8^ m/s R (NaCl) = 88.1%	[72]
15.5% PSF/0.5% PVP/3% LiCl 1% MPD/0.05% TMC 0.5% zeolite in dope solution	DI water	2M NaCl	—	0.5 L/min	40	29	86	57	A = 3.3 LMH/bar R (NaCl) = 91.3% S = 340 μm	[73]
15.5% PSF/2% PVP 2% MPD/0.1% TMC 0.4% zeolite in dope solution	10 mM NaCl	2M NaCl	room	0.8 L/min	24.61	14.6	33.1	20	A = 6.86 × 10^−12^ m/s.Pa B = 9.6 × 10^−8^ m/s R (NaCl) = 94.7% S = 480 μm	[74]
16.5% PSF/0.5% PVP 2% MPD/0.15% TMC 0.6% TiO_2_ in dope solution	DI water	2M NaCl	ambient	0.35 L/min	33	15.7	59.4	31	A = 7.3 × 10^−12^ m/s.Pa B = 12.4 × 10^−8^ m/s R (NaCl) = 93.6% S = 390 μm	[75]
17.41% PSF/0.5% PVP 2% MPD/0.1% TMC 0.5% TiO_2_ in dope solution	10 mM NaCl	2M NaCl	ambient	32.72 cm/s	29.7	7.3	56.27	14.14	A = 5.45 × 10^−12^ m/s.Pa B = 10.66 × 10^−8^ m/s R (NaCl) = 92.7% S = 420 μm	[76]
16% PSF 2% MPD/2% TEA/0.2% TMC 0.1% TiO_2_ in aqueous solution	10 mM NaCl	2M NaCl	—	0.8 L/min	40	12.3	26	13	A = 12.26 × 10^−12^ m/s.Pa B = 49.9 × 10^−8^ m/s R (NaCl) = 86% S = 650 μm	[77]
16% PEF/1% PVP/2.5% PEG-200 2% MPD/0.1% TMC 0.05% SiO_2_ in aqueous solution	DI water	2M NaCl	30 °C	10 L/h	15.22	7.53	23.93	16.15	A = 3.10 LMH/bar B = 0.31 LMH R (NaCl) = 91% S = 362 μm	[78]
16% PSF/1% PVP 2% MPD/0.1% TMC 0.05% SiO_2_ in aqueous solution	10 mM NaCl	2M NaCl	30 °C	0.8 L/min	15	1.6	25.28	3.44	A = 9.52 × 10^−12^ m/s.Pa B = 28.4 × 10^−8^ m/s R (NaCl) = 89% S = 368 μm	[79]
14% PSF/0.5% PVP 1% MPD/0.05% TMC 1% SiO_2_ in dope solution	DI water	2M NaCl	25 °C	0.25 L/min	14.60	9.00	23.50	20.06	A = 2.96 × 10^−12^ m/s.Pa B = 4.79 × 10^−8^ m/s R (NaCl) = 86.18%	[80]
E.Spun N6/20% SiO_2_ 1% MPD/0.15% TMC 4% SiO_2_ in aqueous solution	DI water	1M NaCl	24 °C	26.3 cm/s	27.10	9.35	—	—	A = 45 LMH/MPa B = 1.24 LMH R (NaCl) = 98.5% S = 365 μm	[81]
14% PSF/ 2%PVP 2% MPD/0.1% TMC 0.5% ZnO in dope solution	10 mM NaCl	2M NaCl	—	—	30.06	17.31	—	—	A = 7.39 × 10^−12^ m/s.Pa B = 20.55 × 10^−8^ m/s R (NaCl) = 89.99% S = 400 μm	[82]
2g PSF 2% MPD/0.1% TMC 0.25% ZnO@PMMA in aqueous solution	DI water	1M NaCl	ambient	0.1 L/min	14.6	2.2	—	—	A = 2.32 LMH/bar B = 0.28 LMH R (NaCl) = 97.7% S = 693 μm	[83]
16% PSF/6% PVP/2% LiCl 2% MPD/0.1% TMC 0.5% Al_2_O_3_ in dope solution 0.05% Al_2_O_3_ in organic solution	DI water	1M NaCl	—	18.5 cm/s	27.6	7.1	—	—	A = 8.43 LMH/bar B = 1.66 LMH	[84]
14% PES/2% PVP 2% MPD/0.1% TMC 0.2% Fe_3_O_4_ in dope solution	10 mM NaCl	2M NaCl	room	0.8 L/min	28.8	14.7	38.08	20.1	A = 8.55 × 10^−12^ m/s.Pa B = 15.6 × 10^−8^ m/s R (NaCl) = 93.2% S = 420 μm	[85]
17.41% PSF/0.5% PVP/0.5% nano-filler 2% MPD/0.1% TMC 0.5% TiO_2_ and 0.5% GO in dope solution	DI water	2M NaCl	—	2.5 cm/s	23.5	2.7	30.5	4.4	A = 1.61 × 10^−12^ m/s.Pa B = 1.44 × 10^−8^ m/s R (NaCl) = 91.1% S = 200 μm	[86]
18% PES/2% PEG-200 2% MPD/0.1% TMC 0.2% ZnO/SiO_2_ in dope solution	DI water	1M NaCl	25 °C	8.3 cm/s	33.5	12.23	50.1	18.22	A = 3.47 LMH/bar B = 4.01 LMH R (NaCl) = 78.6% S = 297 μm	[87]
18% PVDF/3% PVP 2% MPD/0.1% TMC 0.75% SiO_2_@MWCNT in dope solution	DI water	1M NaCl	—	0.3 L/min	22.1	4.1	28.6	8.05	A = 1.21 LMH/bar B = 0.12 LMH R (NaCl) = 93.6% S = 240.5 μm	[88]
14% PES/2% PVP 2% MPD/0.1% TMC 0.2% Fe_3_O_4_/ZnO in aqueous solution	10 mM NaCl	2M NaCl	23 °C	0.8 L/min	29.3	5.6	—	—	A = 8.24 × 10^−12^ m/s.Pa B = 7.88 × 10^−8^ m/sR (NaCl) = 98.5%S = 400 μm	[89]

**Table 2:** Overview of the physicochemical characteristics of draw agents utilized in FO applications.

Type of Membrane	Type of Draw Agent	Concentration	Osmotic Pressure	Configuration	FO Performance		Regeneration Methods	Application	Advantages	Disadvantages	Reference
					Jw; LMH	Js; gMH					
CA	Polyacrylic acid sodium salt (PAA-Na (Mw = 1200))	0.72 g/mL	44 atm	AL–DS	22	0.17	UF	Wastewater treatment	High water flux, low RSF, and high water solubility	High viscosity, low solute diffusion, expensive precursors, and UF requires energy	[10]
CTA	Sodium alginate sulfonate	600 gNaLS/kg	78 bar	AL–FS AL–DS	8.5 15	12.3 27	NF	Desert Restoration	High osmotic pressure	Limited applications and relatively low water flux	[11]
TFC	Na_3_PO_4_	0.2 M	580 mOsm/kg	AL–FS AL–DS	9.02 16.2	0.95 1.3	MD	Activated sludge	Low RSF and high water solubility	Relatively low FO performances, complicated and energy-intensive recovery	[22]
CTA	EDTA sodium (pH = 8)	0.8 M	—	AL–DS	12.9	0.32	NF	Sludge dewatering	Low energy consumption, high water flux, and low RSF	Expensive solute and low solute rejection with NF recovery	[25]
CTA–non-woven	MgCl_2_	1 M	—	AL–FS	6.3	—	—	Secondary treated effluents	High water flux and high rejection for nutrients up to 97%	High viscosity, high ICP, low diffusion coefficient, and contain scale precursor ions (${\mathrm{Mg}}^{2+}$)	[26]
CTA–woven	Triethylenetetramine hexapropionic acid sodium (TTHP-Na (pH = 8))	0.5 g/mL	165 bar	AL–FS AL–DS	12.87 23.07	0.7 0.75	NF	Dye wastewater treatment	High osmotic pressure and average water flux	NF requires energy	[47]
CTA	KHCO_3_	1.4 M	2.8 MPa	AL–FS	5.54	1.2	RO	Desalination	Low RSF	Limited applications, and contain scale precursor ions (${\mathrm{CO}}_{3}^{2-}$) and not easily recovered by RO	[102]
CTA	KBr	0.6 M	2.8 MPa	AL–FS	10.22	22	RO	Desalination	—	Very high RSF and high replenishment cost	[102]
CTA–nylon mesh	NH_4_HCO_3_	3.6 M	—	AL–FS AL–DS	7 9	—	Moderate heating	Desalination	High osmotic pressure capable of desalinating seawater and high FO performances	Low solubility and ammonia smell in water, high RSF, contain scale precursor ions (${\mathrm{CO}}_{3}^{2-}$), high replenishment cost, and not thermally stable	[105]
CTA–polyester screen	NaCl	0.6 M	27.44 bar	AL–FS	6.3	7.25	—	Desalination	High osmotic pressure, high solubility, low viscosity, and low cost	High RSF and high fouling tendency	[106]
CTA–polyester screen	NaHCO_3_	0.72 M	26.91 bar	AL–FS	5.81	2.85	—	Desalination	Low cost	Low water solubility and contain scale precursor ions (${\mathrm{CO}}_{3}^{2-}$)	[106]
CTA	KCl	2 M	89.3 bar	AL–FS	15.2	26.8	Direct fertigation	Fertilizer	High osmotic pressure, high solubility, low viscosity, and low cost	High RSF and high fouling tendency	[107]
TFC–polyamide	NH_4_Cl	0.5 M	21.881 atm	AL–FS AL–DS	9.87 15.37	—	—	Desalination	Diluted draw solution could be directly used in irrigation	High RSF	[108]
TFC–polyamide	NaNO_3_	0.5 M	21.3 atm	AL–FS AL–DS	7.97 14.26	—	—	Desalination	Diluted draw solution could be directly used in irrigation	High RSF and high biofouling tendency	[108]
TFC–polyamide	KNO_3_	0.5 M	20.125 atm	AL–FS AL–DS	9 13.83	—	—	Desalination	Diluted draw solution could be directly used in irrigation	High RSF, high biofouling tendency, toxic and energy-intensive	[108]
TFC–polyamide	NH_4_NO_3_	0.5 M	17.764 atm	AL–FS AL–DS	7.9 11.88	—	—	Desalination	Diluted draw solution could be directly used in irrigation	High RSF and contain scale precursor ions (${\mathrm{CO}}_{3}^{2-}$)	[108]
TFC–polyamide	Ca(NO_3_)_2_	0.5 M	26.491 atm	AL–FS AL–DS	8.21 15.41	—	—	Desalination	Diluted draw solution could be directly used in irrigation	Contain scale precursor ions (${\mathrm{Ca}}^{2+}$), high replenishment cost, and poor water extraction capacity	[108]
TFC–polyamide	CaCl_2_	0.5 M	34.983 atm	AL–FS AL–DS	8.8 16.32	—	—	Desalination	High water flux and diluted draw solution could be directly used in irrigation	High RSF and contains scale precursor ions (${\mathrm{Ca}}^{2+}$)	[108]
TFC	Na_2_SO_4_	1 M	—	AL–FS AL–DS	15.7 23.26	4.9 7.1	—	Desalination	High water flux	High RSF and contain scale precursor ions (${\mathrm{SO}}_{4}^{2-}$)	[109]
TFC–polyamide	MgSO_4_	1 M	—	AL–FS	11	1.32	Direct fertigation	Fertilizer	Higher diffusivity and does not require energy for recovery	Low FO performances, high viscosity, low water solubility, contain scale precursor ions (${\mathrm{SO}}_{4}^{2-}$) and reaction products are toxic and expensive reagents	[110]
TFC–polyamide	Mg(NO_3_)_2_	1 M	84 bar	AL–FS	30.8	24.18	Direct fertigation	Fertilizer	High osmotic pressure, high water flux, and does not require energy for recovery	High RSF	[110]
CA–polyester woven	(NH_4_)_2_SO_4_	2 M	92.1 atm	AL–FS	19.41	2.6	Direct fertigation	Fertilizer	High osmotic pressure and does not require energy for recovery	Contains scale precursor ions (${\mathrm{SO}}_{4}^{2-}$) and high replenishment cost	[111]
CA–polyester woven	NH_4_H_2_PO_4_	2 M	86.3 atm	AL–FS	16.65	28.7	Direct fertigation	Fertilizer	Diluted draw solution could be directly used	Low water flux and high biofouling tendency	[111]
CA–polyester woven	(NH_4_)_2_HPO_4_	2 M	95 atm	AL–FS	14.01	4.6	Direct fertigation	Fertilizer	Diluted draw solution could be directly used	Low water flux and high biofouling tendency	[111]
CTA	NaH_2_PO_4_	22 g/L	23.73 bar	AL–FS	2.63	0.12	Direct fertigation	Fertilizer	Low RSF and does not require energy for recovery	Low water flux	[112]
CA	Sucrose	1 M	26.7 atm	AL–FS	12.9	—	NF	Wastewater treatment	Large molecule and high water solubility	Low osmotic pressure, low water flux, NF requires energy and relatively low FO performances	[113]
CA	Glucose	—	—	—	—	—	—	—	Large molecule and high water solubility	Low osmotic pressure, high ICP effect, and used only for emergency water supply	[114]
TFC	Poly(maleic acid) sodium (PMAS)	0.5 mol/kg	143 bar	AL–FS AL–DS	23.5 30.6	0.6 0.68	NF	Desalination	High osmotic pressure, big molecular size, high FO performance, and negligible RSF	NF requires energy	[115]
TFC–polyamide	Cobaltic complex (Na-Co-CA)	1 M	—	AL–FS	11.5	—	MD or NF	Heavy metal wastewater treatment	High osmotic pressure, high water flux, low RSF, low replenishment cost, minimize TDS in FS, and efficiency of waste water treatment	Recovery requires energy	[116]
CA	Cu complex (Cu-CA)	1 M	—	AL–FS AL–DS	8.53 15.16	0.08 0.11	—	Seawater desalination	High FO performance and negligible RSF	Complicated preparation	[117]
CA	Fe complex (Fe-CA)	1 M	—	AL–FS AL–DS	10.78 21	0.12 0.14	—	Seawater desalination	High FO performance and negligible RSF	Complicated preparation	[117]
TFC	Poly(aspartic acid sodium salt) (PAsp-Na)	0.3 g/mL	51.5 atm	AL–FS AL–DS	8.13 16.62	1.64 2.28	MD and NF	Wastewater reclamation Brackish water desalination	Low RSF, inhibits the scaling formation, and nontoxic	Average FO performances and recovery require energy	[118]
TFC	Polyamidoamine with terminal carboxyl groups (PAMAM-COONa (2.5 G))	0.5 g/mL	3603 mOsm/kg	AL–DS	29.7	7.5	MD	Wastewater treatment and protein enrichment	High osmotic pressure, low viscosity, relatively large molecular size, and low RSF	Low water flux tested only in AL–DS mode and not feasible	[119]
TFC	Poly(sodium4-styrene sulfonate) (PSS (Mw = 1200))	0.24 g/mL	—	AL–DS	18.2	5.5	UF	—	High osmotic pressure, low viscosity and high water flux	High RSF, lower diffusion coefficient, more severe CP, 40% water flux reduces after the regeneration and requires energy for UF	[120]
CTA	Poly(isobutylene-alt-maleic acid) sodium salt (PIMA-Na)	0.375 g/mL	—	AL–DS	34	0.196	MD	Seawater desalination	Low RSF and nontoxic	Relatively low FO performances, high viscosity, and low water flux when tested with seawater	[121]
CTA	Thermo-responsive PNIPAM/γ-PGA/PEG hydrogel	—	—	AL–FS	1.99	—	Heat in a water bath at 40 °C	Desalination	Low energy consumption and negligible RSF	Poor water flux	[122]
CTA-polyester mesh	Electric-responsive HA/PVA hydrogel (6 V)	—	—	AL–FS	25.49	—	Electric field at 6 V	Desalination	High water flux, negligible RSF, and more safe and efficient when regenerating drinking water	___	[123]

**Table 3:** Effect of operating conditions on FO performance in the literature.

**The Effect of DS and FS Flow Rate on the Performance of the FO Process**						
**Type of Membrane**	**Temperature**	**Feed Solution**	**Draw Solution**	**Flow Rate**	**Water Flux**	**Reference**
CTA flat sheet	25 °C	DI water	0.3 M EDTA-Na	62–384 cm/min counter-current mode	6.13–7.12 LMH (PRO mode)	[25]
CTA flat sheet	25 °C	Nutrients	1 M MgCl_2_	0.5–1 L/min counter-current mode	6.3–11.3 LMH (FO mode)	[26]
CTA flat sheet	25 ± 2 °C	Seawater	200 g/L multicomponent fertilizer	1.6–3.2 L/min in FS 1.6 L/min in DS counter-current mode	9.63–9.87 LMH (FO mode)	[112]
CTA flat sheet	25 ± 2 °C	Seawater	200 g/L multicomponent fertilizer	1.6–2.4 L/min in DS 1.6 L/min in FS counter-current mode	9.63–8.87 LMH (FO mode)	[112]
TFC flat sheet	25 ± 1 °C	0.1 M NaCl	0.6 M NaCl	0.4–0.8 L/min counter-current mode	6.85–7.21 LMH (FO mode)	[135]
TFC flat sheet	20 °C	Distilled water	0.5 M NaCl	1.2–3.4 L/min counter-current mode	27.5–42 LMH (PRO mode)	[136]
TFC flat sheet	20 °C	Distilled water	0.5 M NaCl	1.2–3.4 L/min in FS 1.2 L/min in DS counter-current mode	27.45–38.02 LMH (PRO mode)	[136]
TFC–ES flat sheet	40 °C	DI water	3.5 wt% NaCl	14.4–48 mL/min co-current mode	5.1–9.4 LMH (PRO mode)	[137]
**The Effect of DS and FS Concentration on the Performance of the FO Process**						
**Type of Membrane**	**Temperature**	**Feed Solution**	**Draw Solution**	**Flow Rate**	**Water Flux**	**Reference**
CTA flat sheet	25 ± 1 °C	DI water	0.1–0.5 M TTHP-Na	0.3 L/min co-current mode	9.38–12.87 LMH (FO mode) 17.64–23.07 LMH (PRO mode)	[8]
CTA flat sheet	25 °C	DI water	0.1–1.0 M EDTA-Na	384 cm/min counter-current mode	4.02–13.08 LMH (PRO mode)	[25]
TFC flat sheet	25 °C	Ethanol	1–4 M LiCl in ethanol	0.2 L/min counter-current mode	1.5–5.6 LMH (FO mode) 2–7.9 LMH (PRO mode)	[29]
TFC flat sheet	25 °C	1000–10,000 ppm Tetracycline	2 M LiCl in ethanol	0.2 L/min counter-current mode	2.7–1 LMH (FO mode)	[29]
TFC flat sheet	25 °C	1000–5000 ppm heavy metal ions	1 M cobaltic complex (Na–Co–CA)	0.2 L/min co-current mode	11.5–10.5 LMH (FO mode)	[116]
CA flat sheet	25 °C	0–8000 ppm NaCl	HA-PVA-5 polymer hydrogels	0.4 L/min	25.49–12.44 LMH (FO mode)	[123]
TFC-ES flat sheet	40 °C	DI water	2.5–7.7 wt% NaCl	48 mL/min co-current mode	7.5–11.4 LMH (PRO mode)	[137]
CTA flat sheet	23 °C	DI water	0.5–4 M NaCl	22.5 cm/s co-current mode	10.1–28.8 LMH (FO mode) 16.9–48.1 LMH (PRO mode)	[138]
CTA flat sheet	25 °C	Brackish water	3–4 M CaCl_2_	8.5 cm/s counter-current mode	12–14 LMH (FO mode)	[139]
TFC hollow fiber	25 °C	DI water	1–4 M NaCl	0.2 L/min co-current mode	18–49 LMH (FO mode)	[140]
**The Effect of DS and FS Temperature on the Performance of the FO Process**						
**Type of Membrane**	**Temperature**	**Feed Solution**	**Draw Solution**	**Flow Rate**	**Water Flux**	**Reference**
CTA flat sheet	25–45 °C	DI water	3 M KCl	0.4 L/min counter-current mode	5.3–7.6 μm/s (FO mode)	[105]
CTA flat sheet	25 ± 2 °C in FS 30–45 °C in DS	Pure water	4M NH_4_HCO_3_	0.15 L/min co-current mode	1.95–2.4 μm/s (FO mode) 2.5–3.17 μm/s (PRO mode)	[105]
TFC flat sheet	25–45 °C	Distilled water	0.6 M NaCl	10 cm/s counter-current mode	6.3–7.14 μm/s (FO mode)	[106]
TFC flat sheet	25 °C in FS 25–60 °C in DS	2000 ppm heavy metal ions	1 M cobaltic complex (Na–Co–CA)	0.2 L/min co-current mode	11–16.5 LMH (FO mode)	[116]
TFC flat sheet	20 °C in FS 20–32 °C in DS	Distilled water	0.5 M NaCl	1.2 L/min counter-current mode	25.1–32.9 LMH (PRO mode)	[136]
TFC flat sheet	20–32 °C in FS 20 °C in DS	Distilled water	0.5 M NaCl	1.2 L/min counter-current mode	26.4–35.6 LMH (PRO mode)	[136]
TFC–ES flat sheet	25 °C in FS 23–60 °C in DS	DI water	3.5 wt% NaCl	48 mL/min co-current mode	9.2–9.8 LMH (PRO mode)	[137]
CTA flat sheet	20 ± 1 °C in FS 20–50 °C in DS	DI water	3 M NaCl	8.5 cm/s co-current mode	18.8–26.8 LMH (FO mode)	[141]
CTA flat sheet	25–45 °C	Salinity	117 g/L NaCl	10 cm/s counter-current mode	14.47–18.82 LMH (FO mode)	[141]
CTA flat sheet	20 ± 1 °C in DS 20–50 °C in FS	DI water	3 M NaCl	8.5 cm/s co-current mode	18.8–27.1 LMH (FO mode)	[142]

**Table 4:** Analysis method of the effect of concentration polarization (CP) on FO performance.

Analysis Method	Feed Solution NaCl (M)	Draw Solution NaCl (M)	Flow Rate (L/min)	Temp. (°C)	Δπ_eff_%	DICP%	DECP%	CECP%	Water Flux (L/m^2^ h)	Reference
Analytical using TFC membrane	0.1	0.6	0.5–1	25	29.66–30.41	46.94–49.97	19–16.02	4.4–3.61	6.98–7.32	[135]
	0.1	0.6	0.5–1 FS/0.5 DS	25	29.66–30.24	46.94–47.16	19–19.14	4.4–3.46	6.98–7.18	
	0.1	0.6	0.5–1 DS/0.5 FS	25	29.66–30.33	46.94–49.4	19–15.73	4.4–4.53	6.98–7.02	
Analytical using TFC membrane	0	1–5	10 cm/s	25	23.5–6.2	39–44.7	21.7–40.3	15.7–8.8	3.55–24.04	[147]
	0	3	5–25 cm/s	25	12.2–13.9	39.2–50	36.7–27.8	12–8.3	13.3–18	
	0	3	10 cm/s	25–45	13.6–17.5	43.7–39.3	32.5–32.9	10.2–10.3	15.25–22.08	
MATLAB software	0	1–4	0.1	–	41.7–19.1	46.8–60.4	11.3–20.4	0.0747–0.154	8.8–17	[153]
	0–3	1–4	0.1	–	41.7–10.7	46.8–67.6	11.3–12.2	0–9.44	9–2.3	
	0	3	0.1–1.7	–	–	46–60.6	32.5–15.5	negligible	13.6–15.1	
	3	6	0.1–1.7	–	–	54.9–71.6	24.3–12.6	13.8–6.76	4.5–5.8	

**Table 5:** Recent studies on factors affecting reverse solute flux (RSF) for the FO process.

Groups	Draw Solution	Reverse Solute Flux	Findings	Reference
Different membrane properties				
Tortuosity (1.07–2.5)	2 M NaCl	0.155–0.1 mol/m^2^h	High tortuosity leads to declining both water flux and RSF since lengthens the mass transfer path and reduces the mass transfer coefficient, which would amplify ICP.	[169]
Porosity (0.15–0.95)	2 M NaCl	0.065–0.18 mol/m^2^h	High porosity (ε) leads to increasing both water flux and RSF since enhances the concentration gradient and reduces the resistance to solute diffusion (i.e., dilutive ICP). When ε > 0.8, the enhancement of water flux becomes less significant but RSF enhancement is still significant. Thus, higher ε does not always mean better performance.	[169]
Pore size (0.025–0.45 nm)	1.5 M NaCl	0.93–8.30 g/m^2^h	The pore size of about 0.2 μm promoted both high water flux and low RSF due to its open, highly porous structure and reduced tortuosity creating less resistance to water transport and solute diffusion (i.e., lower S value = 1220 ± 380). It also helped the selective layer to avoid defects, resulting in a higher cross-linking degree and hence higher selectivity.	[170]
Different salt solutions with the same ion				
Na^+^	0.6 M NaCl 0.72 M NaHCO_3_	8.17 g/m^2^h 3.22 g/m^2^h	NaCl is higher in water flux and 2.5 times larger than NaHCO_3_ in reverse diffusion. Although identical in the osmotic pressure (28 bar) and the presence of ${\mathrm{Na}}^{+}$ in both solutions, the size of the hydrated anion is what causes this difference, i.e., ${\mathrm{HCO}}_{3}^{–1}$ (0.45 nm) > ${\mathrm{Cl}}^{-}$ (0.3 nm).	[106]
Mg^2+^	1 M MgSO_4_ 1 M Mg(NO_3_)_2_	1.32 g/m^2^h 24.18 g/m^2^h	${\mathrm{Mg}}^{2+}$ is completely soluble in water as Mg(NO_3_)_2_ produces the highest osmotic pressure (84 bar at 1 M) and the highest diffusion (3.31 × 10^−6^ m^2^/h) (i.e., reducing dilute ICP) this will ensure three times higher water flux compared to MgSO_4_ (1.7 × 10^−6^ m^2^/h). Thus, RSF typically increases as water flux increases.	[110]
Different ions	22 g/L NH_4_Cl 22 g/L KCl 22 g/L (NH_4_)_2_SO_4_ 22 g/L NaH_2_PO_4_	3.71 g/m^2^h 1.98 g/m^2^h 0.82 g/m^2^h 0.12 g/m^2^h	${\mathrm{NH}}_{4}^{+}$ showed the highest RSF, followed by $K^{+}$, ${\mathrm{SO}}_{4}^{2-}$ and ${\mathrm{PO}}_{4}^{3-}$. It has been noted that cations paired with ${\mathrm{Cl}}^{-}$ anion have high RSF than those that pair with the sulfate group. While, multivalent negatively charged anions, such as ${\mathrm{SO}}_{4}^{2-}$ and ${\mathrm{PO}}_{4}^{3-}$, have RSF lower than that of monovalent anions because of higher electrostatic repulsion via the negatively charged CTA membrane. Then, KCl exhibited the highest water flux followed by NH_4_Cl, (NH_4_)_2_SO_4_, and NaH_2_PO_4_.	[112]
Different operating conditions				
Concentration	0.5–3 M CaCl_2_	2.55–11.45 g/m^2^h	Increased viscosity and osmotic pressure, and low diffusion coefficient are all effects of higher DS concentration, which also increases water flux and RSF, but it will not be beneficial as it may cause FS contamination.	[139]
Flow rate	1 M NaCl at 0.2–1 L/min	4.3–2.8 g/m^2^h	Decreased concentrative ECP and increased dilutive ICP are all effects of a higher DS flow rate, which results in decreases in the water flux and RSF since it reduced the residence time of liquid in the FO unit.	[140]
Temperature	3 M NaCl at 20–50 °C	0.21–0.3 mol/m^2^h	Reduced viscosity (1.3408–0.7574 mPa.s), CP, increased osmotic pressure (162.95–173.61 bar), diffusion coefficient (1.067–2.063 nm^2^/s), and water permeability are all effects of higher temperature, which also increases the water flux and RSF. However, it may raise the risk of membrane fouling brought on by an increase in ion permeability and membrane clogging (i.e., larger hydrated ion size).	[142]
Different nanoparticles (NPs)				
SiO_2_ (negative), TiO_2_ (neutral) and ZnO (positive) in feed solution	0.5 M NaCl	16.8 mol/m^2^h 16.5 mol/m^2^h 15.7 mol/m^2^h	ZnO (29.7 mV) and TiO_2_ (0.6 mV) showed higher RSF because they carried a positive charge opposite to the membrane charge (−12 mV), which forms a fouling layer on the surface that attracts ions in the DS and impedes water flux. Whereas SiO_2_ (−20.2 mV) formed a relatively thin film of fouling, which facilitates water transport. After the aggregation of NPs with NaCl for 30 min, a size increase in less than 20% was observed for SiO_2_ (42–49 nm) and ZnO (41–50 nm). While it increases by 40% for TiO_2_ (38–54 nm). Thus, the aggregation of NPs may not significantly impact FO performance.	[171]
TiO_2_ and Al_2_O_3_ in the support layer	1 M NaCl	7. 1 g/m^2^h 5.4 g/m^2^h	1% TiO_2_ in the support layer leads to high water flux and lower RSF due to increased porosity and hydrophilicity (80.72%, 61.85°) compared to the CA membrane (71.81, 67.86°). However, we notice a further decrease in RSF by adding 0.1% Al_2_O_3_ to the TiO_2_-modified membrane (80.96%, 56.7°). However, a further increase in NPs loading can lead to lower water flux and higher RSF due to NPs aggregation in the sublayer.	[172]
GO in the active layer	1 M NaCl	2.6 g/m^2^h	0.1% GO in the active layer leads to high water flux and lower RSF due to increased roughness and hydrophilicity (54.1 nm, 64°) compared to the control membrane (31.7 nm, 82°). However, a further increase in GO loading leads to agglomeration of the nanostructure, which limits the formation of the ideal thin film of the polyamide layer and consequently to lower water flux and higher RSF.	[173]

## Data Availability

Not applicable.
